# The repertoire and structure of adhesion GPCR transcript variants assembled from publicly available deep-sequenced human samples

**DOI:** 10.1093/nar/gkae145

**Published:** 2024-02-29

**Authors:** Christina Katharina Kuhn, Udo Stenzel, Sandra Berndt, Ines Liebscher, Torsten Schöneberg, Susanne Horn

**Affiliations:** Rudolf Schönheimer Institute of Biochemistry, Medical Faculty, University of Leipzig, 04103 Leipzig, Germany; Rudolf Schönheimer Institute of Biochemistry, Medical Faculty, University of Leipzig, 04103 Leipzig, Germany; Rudolf Schönheimer Institute of Biochemistry, Medical Faculty, University of Leipzig, 04103 Leipzig, Germany; Rudolf Schönheimer Institute of Biochemistry, Medical Faculty, University of Leipzig, 04103 Leipzig, Germany; Rudolf Schönheimer Institute of Biochemistry, Medical Faculty, University of Leipzig, 04103 Leipzig, Germany; Department of Biochemistry, School of Medicine, University of Global Health Equity (UGHE), PO Box 6955 Kigali, Rwanda; Rudolf Schönheimer Institute of Biochemistry, Medical Faculty, University of Leipzig, 04103 Leipzig, Germany; Institute of Translational Genomics, Helmholtz Zentrum München - German Research Center for Environmental Health, 85764 Neuherberg, Germany

## Abstract

Alternative splicing and multiple transcription start and termination sites can produce a diverse repertoire of mRNA transcript variants from a given gene. While the full picture of the human transcriptome is still incomplete, publicly available RNA datasets have enabled the assembly of transcripts. Using publicly available deep sequencing data from 927 human samples across 48 tissues, we quantified known and new transcript variants, provide an interactive, browser-based application *Splice-O-Mat* and demonstrate its relevance using adhesion G protein-coupled receptors (aGPCRs) as an example. On average, 24 different transcript variants were detected for each of the 33 human aGPCR genes, and several dominant transcript variants were not yet annotated. Variable transcription starts and complex exon-intron structures encode a flexible protein domain architecture of the N- and C termini and the seven-transmembrane helix domain (7TMD). Notably, we discovered the first GPCR (ADGRG7/GPR128) with eight transmembrane helices. Both the N- and C terminus of this aGPCR were intracellularly oriented, anchoring the N terminus in the plasma membrane. Moreover, the assessment of tissue-specific transcript variants, also for other gene classes, in our application may change the evaluation of disease-causing mutations, as their position in different transcript variants may explain tissue-specific phenotypes.

## Graphical Abstract

**Graphical Abstract:**
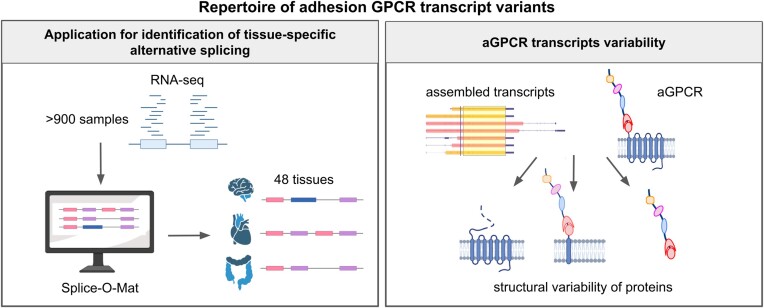

## Introduction

Plasma membrane receptors scan the cellular environment for information and integrate various chemical and physical signals into intracellular responses. Among membrane receptors, the superfamily of G protein-coupled receptors (GPCRs) includes over 800 members in the human genome, most belonging to the class of rhodopsin-like receptors (class A) (1). Approximately half of the GPCR genes have a single protein-coding exon (2) leaving only few options to express multiple receptor protein isoforms. However, the class of adhesion GPCR (aGPCR) genes is unique among GPCRs, because most encode for large proteins with up to ∼6,300 amino acid residues (ADGRV1, ∼700 kDa) (3). These proteins are all derived from genes with complex exon-intron structures and, therefore, spliced mRNA, which are not entirely elucidated yet. We have previously shown that mouse aGPCR genes express, on average, 19 different transcript variants (4). These findings have implications on the domain architecture of aGPCR proteins, their functional properties, the rationales for design of aGPCR constructs used for structural analysis [X-ray crystallography, cryogenic electron microscopy (cryo-EM)], and the generation of gene-deficient mouse lines.

As all GPCRs, aGPCRs contain a seven-transmembrane helix domain (7TMD) which transduces extracellular signals and mediates intracellular G-protein coupling via conformational changes. Specifically, the long N terminus of aGPCRs receives extracellular signals, such as ligand binding or mechanical forces, which are transmitted via an intramolecular agonist, the so-called *Stachel* sequence (5,6), and lead to structural changes within the 7TMD. Furthermore, some aGPCRs have extremely long C termini with yet unknown functional relevance. The long N termini are usually present with a modular domain architecture which can include e.g., pentraxin domains, leucine-rich domains and immunoglobulin domains (7). Many aGPCRs are cleaved by autoproteolysis at the so-called G protein-receptor proteolytic site (GPS) within the GPCR autoproteolysis-inducing (GAIN) domain (8), both located in the N terminus in close proximity to the 7TMD. This autoproteolysis generates an N-terminal fragment (NTF) and a C-terminal fragment (CTF). Currently, most experimental work on aGPCRs is performed on the first annotated transcript variant, not considering tissue-specific splicing and transcript variants generated by different promoters. However, such non-canonical transcript variants can sometimes be more abundant compared to the canonical, annotated transcript variant given as references in sequence databases (4). Our work and others has shown that the annotation of aGPCR transcript variants requires deep-sequencing efforts to cover the full transcript variant repertoire in a given tissue (4,9). However, a comprehensive qualitative and quantitative analysis of all human aGPCRs across various tissues is still lacking.

High coverage sequenced transcriptome data allow for quantitative expression analysis of a given gene, and can also be used for computational reconstruction and quantification of transcript variants. Bioinformatic pipelines for transcriptome analysis usually include splice-aware mapping tools such as STAR (10) or HISAT2 (11), and a transcript assembly and quantification step performed by StringTie (12) or Cufflinks (13,14). Using StringTie, we have recently shown that increasing sequence coverage of the analyzed transcriptome led to a saturation of identified transcripts (4). However, this approach was designed as a proof-of-principle for a limited number of aGPCRs from mouse tissues and lacked an interactive application. Hence, we broadened the analysis to a genome-wide approach based on human data.

Here, we present a qualitative and quantitative analysis of all aGPCR gene-derived transcript variants and an interactive application at https://tools.hornlab.org/Splice-O-Mat/ allowing genome-wide, qualitative and quantitative analysis of transcript variants in 48 human tissues from publicly available high-throughput RNA-Sequencing (RNA-Seq) data. We aimed to extend beyond available and commonly used databases, such as NCBI gene, Ensembl Canonical transcript, UCSC genome browser, and GTEx, by adding comprehensive data on previously unrecognized transcript variants. As aGPCR genes exhibit all known mechanisms of vertebrate transcript variant generation including alternative splicing events (e.g., alternative donor or acceptor sites, exon skipping, cryptic intron usage), variable 5′ promoters, internal (in-gene) promoters, and chimeric transcripts, we demonstrate the power and the relevance of analyzing transcript variants with our application based on this gene class. We specifically asked how many transcript variants aGPCRs have, what exon compositions they contain, and whether there are abundant human transcript variants that have not been annotated yet. We also investigated whether aGPCR genes have internal promoters that could lead to different proteins, such as N-terminally truncated proteins, and whether variations in the exon composition affect the resulting receptor protein structure. This led to the discovery of the first GPCR, ADGRG7/GPR128, with eight transmembrane helices. Furthermore, we evaluated whether transcript variant composition can vary between tissues. All of this has implications not only for understanding aGPCR functions, but also for interpreting clinically relevant genomic changes.

## Materials and methods

### Data processing and transcript assembly

An overview of the bioinformatic workflow is given in Supplementary Figure S1. We obtained publicly available gene expression datasets with RNA-Seq data for brain tissue (GSE173955, GSE182321, GSE101521), one liver tissue dataset (GSE174478), one heart tissue dataset (GSE165303), one kidney tissue dataset (GSE217427), and one dataset including 45 different tissues types (GSE138734) from the Gene Expression Omnibus (GEO, https://www.ncbi.nlm.nih.gov/gds/) (Supplementary Table S1). We further obtained one melanoma cancer dataset (PRJEB23709) from the European Nucleotide Archive (https://www.ebi.ac.uk/ena/browser/). Only paired-end RNA-Seq samples were included and datasets which were obviously generated without random primers were excluded. This resulted in 48 different tissue types with 927 samples (Supplementary Table S2).

The human reference genome (GRCh38) and its annotation were obtained from NCBI (ftp://ftp.ncbi.nlm.nih.gov/genomes/all/GCA/000/001/405/GCA_000001405.15_GRCh38/seqs_for_alignment_pipelines.ucsc_ids/) and UCSC (ftp://hgdownload.soe.ucsc.edu/goldenPath/hg38/bigZips/genes/hg38.ncbiRefSeq.gtf.gz), respectively. The raw data was mapped to GRCh38 using STAR (version 2.7.6a) (10) with default parameters. After sorting, the mapped reads were assembled into transcripts and quantified by StringTie (version v2.1.3b) according to the manual (12), using the annotation as initial set of transcript variants. As established in (4), StringTie parameters ‘read coverage’ (-c), ‘transcript length’ (-m) and ‘bases on both sides of a junction a spliced read has to cover’ (-a) were set to minimal values to avoid missing transcript variants. The parameter ‘fraction of most abundant transcript at one locus’ (-f) was lowered from default (0.01) to 0. Default values were used for all other StringTie parameters. To generate a global, unified set of transcripts across RNA-Seq samples, StringTie merge mode was used providing the reference annotation (-G). All potentially new transcripts, which are not annotated in the reference genome (GRCh38 from NCBI) are referred to as ‘NSTRG’ throughout the manuscript. NSTRGs were assigned to the gene (referred to as ‘gene_name’) derived from the annotated transcript variants with which they overlapped the most. This was done by counting overlapping exonic bases.

For visualization, the longest open reading frame (ORF) was identified for each transcript variant and translated to the protein sequence with the python package Biopython (version 1.80) (15). The transcript variant with the longest protein sequence was then screened for protein domains with InterProScan (version 5.60–92.0) (16) in the Pfam database (-appl).

### Application implementation and user options

Data derived from StringTie were fed into an SQLite database (version 3.39.3, https://bitbucket.org/ustenzel/stringtiedb). An interactive browser-based application was implemented in python (version 3.7), using dash (version 2.6.2) and deployed with the green unicorn server (version 20.1.0) and is available at https://tools.hornlab.org/Splice-O-Mat/. Several interactive functions of the application are implemented: the user can select tissues and a gene of interest. After running the analysis, figures, such as projections of exons and transcript variants onto the genome and locations of predicted protein domains, or heatmaps of relative and absolute transcript variant expression across tissues, and tables are shown and can be downloaded. Tables provide the assembled transcript variants with its annotated gene name (if available), genomic positions of the exons contributing to transcript variants, quantitative measures (TPM, fraction of all transcripts) and custom statistical comparisons of tissue-specific expression of the identified transcript variants. Furthermore, single nucleotide polymorphisms and disease-/phenotype-causing mutations can be projected onto the transcript exon structure.

### Protein sequence analysis

Signal peptide sequences were predicted by SignalP 6.0 (https://services.healthtech.dtu.dk/services/SignalP-6.0/) (17). SMART (http://smart.embl-heidelberg.de) was used for predicting protein domains and signal peptide sequences (18). The transmembrane topology was predicted using TMHMM-2.0 (https://services.healthtech.dtu.dk/services/TMHMM-2.0/) (19), DeepTMHMM (https://services.healthtech.dtu.dk/service.php?DeepTMHMM) and SMART. Protein sequences were aligned and evaluated with Uniprot Ugene (20). The 3D structure of *ADGRG7* was predicted from its amino acid sequence using AlphaFold (21). Disease-causing mutations in *ADGRC1* were extracted from the Human Gene Mutation Database (HGMD®) (22).

### Comparison with diagnostic exome sequencing

We tested if our approach would cover genetic regions that have so far been underrepresented by sequencing approaches. As new transcript variants may play a role in human genetic diagnostics, we compared, as an example, the exon positions of the predicted *CELSR1/ADGRC1* transcript variants with those covered by current diagnostic exome sequencing (Twist Bioscience, https://www.twistbioscience.com/resources/data-files/twist-exome-20-bed-files, accessed June 2023) and the Genome Aggregation Database (GnomAD v.3.2.1, comprehensive gene annotation from GENCODEv35, https://www.gencodegenes.org/human/release_35.html).

### Functional analyses of ADGRG7/GPR128

#### Cloning of ADGRG7/GPR128

The mouse *ADGRG7/GPR128* cDNA (NM_172825.3) was cloned from an intestine cDNA library by RT-PCR, ligated into the mammalian expression vector pcDps (23), and an N-terminal hemagglutinin (HA) epitope tag following the start ATG and a C-terminal FLAG epitope tag were introduced via PCR-based site-directed mutagenesis. Mutant variants were generated by site-directed mutagenesis of the wild-type *GPR128/ADGRG7* construct. All constructs were verified by sequencing.

#### Cell culture, transfection and ELISA

COS-7 cells (ATCC) were grown in DMEM supplemented with 10% fetal bovine serum (FBS), supplemented with 100 U/ml penicillin, and 100 mg/ml streptomycin at 37°C in a humidified incubator with 5% CO_2_. COS-7 cells were split into multiwell plates, and 24 h later, cells were transfected with 500 ng receptor-encoding plasmid DNA/well using Lipofectamine2000 (ThermoFisher) according to the manufacturer's protocol.

To estimate cell surface expression of ADGRG7/GPR128 carrying an N-terminal HA tag, we used an indirect cellular enzyme-linked immunosorbent assay (ELISA) (24). To assess the amounts of full-length HA/FLAG double-tagged ADGRG7/GPR128 constructs and to demonstrate that the reduction of cell surface expression levels is not due to a decrease in receptor expression in general, a sandwich ELISA was used and performed in transiently transfected COS-7 cells as described previously (25).

For immunofluorescence studies, cells were seeded in poly-l-lysine treated black Greiner 48-well plates with clear bottom (Greiner, No 655090). Forty-eight hours post-transfection, cells were fixed with 4% formaldehyde in PBS. Permeabilized (0.5% Triton X-100 in PBS) and non-permeabilized cells were blocked with 10% FBS in PBS and incubated with the appropriate tag-specific monoclonal antibodies (anti-HA (Sigma, H3663), 1:400; anti-FLAG (Sigma, F1804) 1:400, in PBS). An anti-mouse IgG-ALEXA Fluor488 conjugate (CiteAB, A11029, 1:500) and DAPI were used to detect the tag-specific antibodies and nuclei, respectively. Images were acquired using the BZ-X810 Fluorescence Microscope, Keyence with a 20x objective.

### Statistical analyses

Spearman correlations and corresponding plots were generated using python and statistical significance was determined using student's tests. Heatmaps were generated by calculating the quantitative contribution of the different transcript variants to the total aGPCR mRNA.

## Results and discussion

### aGPCR sequence analysis

Initially, we tested whether the results of our reference-guided transcript assembly matched the publicly available human transcript variants annotated in NCBI (GRCh38) and given in GTEx (version 8, Supplementary Table S3). Our analysis identified previously known human transcript variants, and the numbers of transcript variants and exons correlated significantly with those annotated in GTEx (Supplementary Figure S2A-B). However, we predicted higher numbers of both transcript variants and exons per gene than found in the publicly available transcript database (Supplementary Figure S2C). Our analysis of all aGPCR genes revealed that 60% (median) of the identified transcript variants were not annotated in the NCBI genome (Supplementary Table S3). Therefore, the majority of the transcript variants found in our study were previously unknown, similar to results from a previous study on mice (4), where also 63% of the transcript variants were new. Notably, some of the human transcript variants newly identified here are the dominant ones, expressed more strongly than the annotated transcript variants, and included transcript variants with differences in the amino acid sequence (e.g., *ADGRC2/CELSR2*).

We further investigated whether the ratio of newly identified transcript variants may be influenced by a specific expression threshold or whether a specific tissue/dataset leads to a high ratio of newly identified transcripts (Supplementary Figure S3A). We did not observe a strong change in the proportion of newly identified transcripts, and even when using a high threshold of included transcripts (expression > 1 TPM), 46% of all identified transcripts are still not annotated. Furthermore, we could not identify any tissue that contributed to a significantly increased number of newly identified transcript variants, and there was no observed relationship between an increased number of samples per tissue and a higher count of newly identified transcript variants (Supplementary Figure S3B).

To compare our tool with studies in mice, we tested whether aGPCR transcript variants previously detected in mouse tissues (4) were also expressed in human tissues. Thus, we compared orthologous human and mouse aGPCR genes (*N* = 18) and found them expressed in human tissue as well with a comparable number of transcript variants and exons identified (Supplementary Table S4). The median of 14.5 (human) versus 15 (mouse) transcript variants assembled from 43 (human) versus 36.5 (mouse) exons indicated a comparable transcript repertoire of aGPCRs across both species which may contribute to future experimental designs (Supplementary Table S4).

### Repertoire of human aGPCR transcript variants

The *Splice-O-Mat* identified transcript variants for all human aGPCR genes (Supplementary Table S5). On average, 24 (median = 11) different transcript variants were derived from each of the human aGPCR genes (*N* = 33) (Table 1). Most transcript variants were found for *ADGRL3/LPHN3* (*N* = 113) and *ADGRG1/GPR56* (*N* = 82) and only a few were found for *ADGRL4/ELTD1* (*N* = 2) and *ADGRF2/GPR111* (*N* = 3). Human aGPCR genes are composed of 46 exons on average (median = 42) with the highest number in *ADGRV1/VLGR1* (129 exons) and *ADGRL3/LPHN3* (99 exons) and the lowest number in *ADGRF4/GPR115* (13 exons), *ADGRG3/GPR97* (14 exons) and *ADGRA1/GPR123* (14 exons).

**Table 1.** Identified aGPCR transcripts. Exon composition of transcript variants for all aGPCRs which were expressed at least in one of the 48 tissues

aGPCR	Old symbol	No. of all transcripts (mean)	No. of 5′-start exons	No. of 3′-end exons	No. of exons (mean per transcript)	No. of all exons in identified transcripts	Median no. of all exons in individual transcripts (min.–max. range)
*ADGRA1*	*GPR123*	6	6	2	6	14	4,5
*ADGRA2*	*GPR124*	5	2	1	18	21	18,4
*ADGRA3*	*GPR125*	11	5	8	32	45	18
*ADGRB1*	*BAI1*	39	25	9	46	80	26
*ADGRB2*	*BAI2*	53	7	7	37	51	30
*ADGRB3*	*BAI3*	7	4	3	35	42	31
*ADGRC1*	*CELSR1*	18	12	11	43	66	34
*ADGRC2*	*CELSR2*	7	4	3	33	40	34
*ADGRC3*	*CELSR3*	11	6	5	84	95	35
*ADGRD1*	*GPR133*	22	10	7	31	48	22,5
*ADGRD2*	*GPR144*	5	5	4	22	31	4
*ADGRE1*	*EMR1*	10	4	4	24	32	18
*ADGRE2*	*EMR2*	72	11	20	44	75	18
*ADGRE3*	*EMR3*	10	5	6	15	26	14
*ADGRE4P*	*EMR4*	10	8	9	19	36	16
*ADGRE5*	*CD97*	5	2	1	21	24	19
*ADGRF1*	*GPR110*	8	3	2	13	18	13
*ADGRF2*	*GPR111*	3	1	3	11	15	10
*ADGRF3*	*GPR113*	24	5	8	23	36	13
*ADGRF4*	*GPR115*	7	4	1	8	13	10
*ADGRF5*	*GPR116*	26	6	5	27	38	22
*ADGRG1*	*GPR56*	82	16	7	33	56	15
*ADGRG2*	*GPR64*	30	9	6	33	48	28
*ADGRG3*	*GPR97*	9	2	1	11	14	11
*ADGRG4*	*GPR112*	12	12	6	26	44	7
*ADGRG5*	*GPR114*	15	7	9	29	45	11
*ADGRG6*	*Gpr126*	19	8	4	32	44	25
*ADGRG7*	*Gpr128*	7	2	3	23	28	15
*ADGRL1*	*LPHN1*	33	10	9	49	68	24
*ADGRL2*	*LPHN2*	79	25	18	39	82	21
*ADGRL3*	*LPHN3*	113	37	15	47	99	24
*ADGRL4*	*ELTD1*	2	1	2	15	18	12
*ADGRV1*	*VLGR1*	37	16	9	104	129	88
	Median	11	6	6	29	42	18

Following the catalog of Halvardson *et al.* (50) identified exons are grouped into 5′ start exons (defined by a donor site), 3′ end exons (defined by an acceptor site) and exons (defined by a donor site and an acceptor site). 5′ start- and 3′-end exons that differ by only ±50 bp were considered as one start- and end exon, respectively.

Different transcription start sites (TSS, first 5′ exon) are indicative for distinct promoters within one gene. Although we considered 5′ start exons with minor differences in the TSS (±50 bp) and with identical 3′ splice donor sites as only one 5′ start exon, an average of 8 potential promoters (median = 6) per aGPCR gene were identified (Table 1). Moreover, many aGPCR genes have internal TSS (in-gene, within an intron) giving rise to N-terminally truncated aGPCR protein isoforms. Interestingly, the GAIN domain with its GPS and the 7TMD of most aGPCR genes are located at different coding exons, and some aGPCRs have TSS within the separating intron or GAIN domain-coding exons, which generate transcripts encoding for CTF-like receptor proteins (Table 2). This has already been suggested from mouse transcriptome data (4). Recent cryo-EM structures of aGPCR-CTF (26–29) were considered artificial because of the missing N terminus, however, our data clearly indicate CTFs as naturally occurring protein isoforms derived from at least some mouse and human aGPCR genes. The termination of transcription and, therefore, the length of the 3′ exon is less variable than the number of the transcripts starting from different TSS. On average, 6 different 3′ exons (median = 6) per aGPCR gene were found (Table 1).

**Table 2.** Putative receptor isoforms derived from mouse aGPCR transcripts

	Old symbol	NTF domain variability	Variability in C terminus	Variability in 7TMD	Soluble N terminus	Membrane anchored N terminus	CTF (or N-terminally truncated)	Signal peptide	Chimeric transcript
ADGRA1	GPR123			✓		✓	✓	0	
ADGRA2	GPR124	✓		✓				1	
ADGRA3	GPR125	✓				✓	✓	2	LOC100505912
ADGRB1	BAI1	✓	✓	✓	✓	✓	✓	1	
ADGRB2	BAI2	✓	✓	✓		✓	✓	2	
ADGRB3	BAI3	✓			✓		✓	1	
ADGRC1	CELSR1	✓	✓		✓		✓	1	
ADGRC2	CELSR2	✓	✓					1	
ADGRC3	CELSR3	✓	✓	✓	✓	✓		2	NCKIPSD, SLC26A6
ADGRD1	GPR133	✓	✓	✓	✓	✓	✓	2	
ADGRD2	GPR144	✓	✓		✓			0*	
ADGRE1	EMR1	✓		✓	✓			1	
ADGRE2	EMR2	✓	✓	✓	✓	✓	✓	1	
ADGRE3	EMR3	✓			✓		✓	1	
ADGRE4P	EMR4				✓			0	
ADGRE5	CD97	✓	✓	✓				1	
ADGRF1	GPR110	✓			✓		✓	1	
ADGRF2	GPR111	✓	✓				✓	1	ADGRF4
ADGRF3	GPR113	✓	✓			✓	✓	2	
ADGRF4	GPR115	✓					✓	1	
ADGRF5	GPR116	✓	✓				✓	1	
ADGRG1	GPR56	✓	✓	✓			✓	2	ADGRG3
ADGRG2	GPR64	✓	✓	✓	✓		✓	1	
ADGRG3	GPR97			✓		✓	✓	1	
ADGRG4	GPR112	✓			✓	✓		1	
ADGRG5	GPR114	✓			✓	✓		1	LOC107984889, ADGRG1
ADGRG6	Gpr126	✓	✓	✓	✓	✓		2	
ADGRG7	Gpr128		✓				✓	1	TFG
ADGRL1	LPHN1	✓	✓		✓	✓	✓	1	ASF1B
ADGRL2	LPHN2	✓	✓	✓				1	
ADGRL3	LPHN3	✓	✓	✓		✓	✓	1	
ADGRL4	ELTD1	✓						1	LOC107984998
ADGRV1	VLGR1	✓	✓		✓	✓		1	
	***%***	87.9	60.6	45.4	51.5	45.4	60.6		

*Signal peptide is missing and there is only a weak prediction for the soluble N terminus

Based on the ORFs of the identified transcripts, the resulting aGPCR proteins were categorized. Categories are described in Figure 2. 7TMD, seven-transmembrane domain; CTF, C-terminal fragment; NTF, N-terminal fragment.

### Adhesion GPCRs show extensive tissue-specific expression of transcript variants

Adhesion GPCRs are abundantly found in most tissues and usually among the highly expressed GPCRs, e.g., in pancreatic islets, adipose tissue, liver, and neurons (30). Our comprehensive analysis of a broad range of tissues revealed that a median of 22 out of 33 aGPCRs were significantly expressed in each tissue investigated (expression > 0.5 TPM, Supplementary Table S6). Nearly all aGPCRs (30 aGPCRs) are expressed in trachea tissue, while only 14 aGPCRs in skeletal muscle. Eight aGPCRs (*ADGRA2, ADGRA3, ADGRF5, ADGRG7, ADGRL1-4*) were expressed in all analyzed tissues. Several aGPCRs were highly expressed in specific tissues, among them *ADGRB2* (brain opioid, 337 TPM), *ADGRF5* (lung, 194 TPM), *ADGRL1* (brain, 190 TPM), *ADGRB1* (brain, 182 TPM), *ADGRL4* (adipose tissue, 171 TPM), and *ADGRG6* (placenta, 162 TPM). In many cases, the high expression corresponds to the described aGPCR function in the respective tissue. For example, ADGRF5/GPR116 is a key regulator of lung surfactant homeostasis (31–33). *ADGRG6/GPR126* has its highest expression in placenta trophoblast giant cells, and gene deficiency leads to placental dysfunction and embryonic lethality (34). ADGRB1, highly expressed in brain tissue and known as brain-specific angiogenesis inhibitors 1, plays an important role for the formation of new synapses between neurons (35).

*ADGRD2* showed tissue-specific expression at low levels (1.46 and 0.98 TPM) in the prostate and thymus, and in parts by transcript variants that do not encode the full-length receptor. One may speculate that *ADGRD2* is expressed only in a very distinct cell population, or is even a pseudogene in humans as in several other mammals e.g., mouse, rat, elephant and orca (36). Other aGPCRs with low expression are *ADGRE3, ADGRF2* and *ADGRG5*.

**Figure 1.**
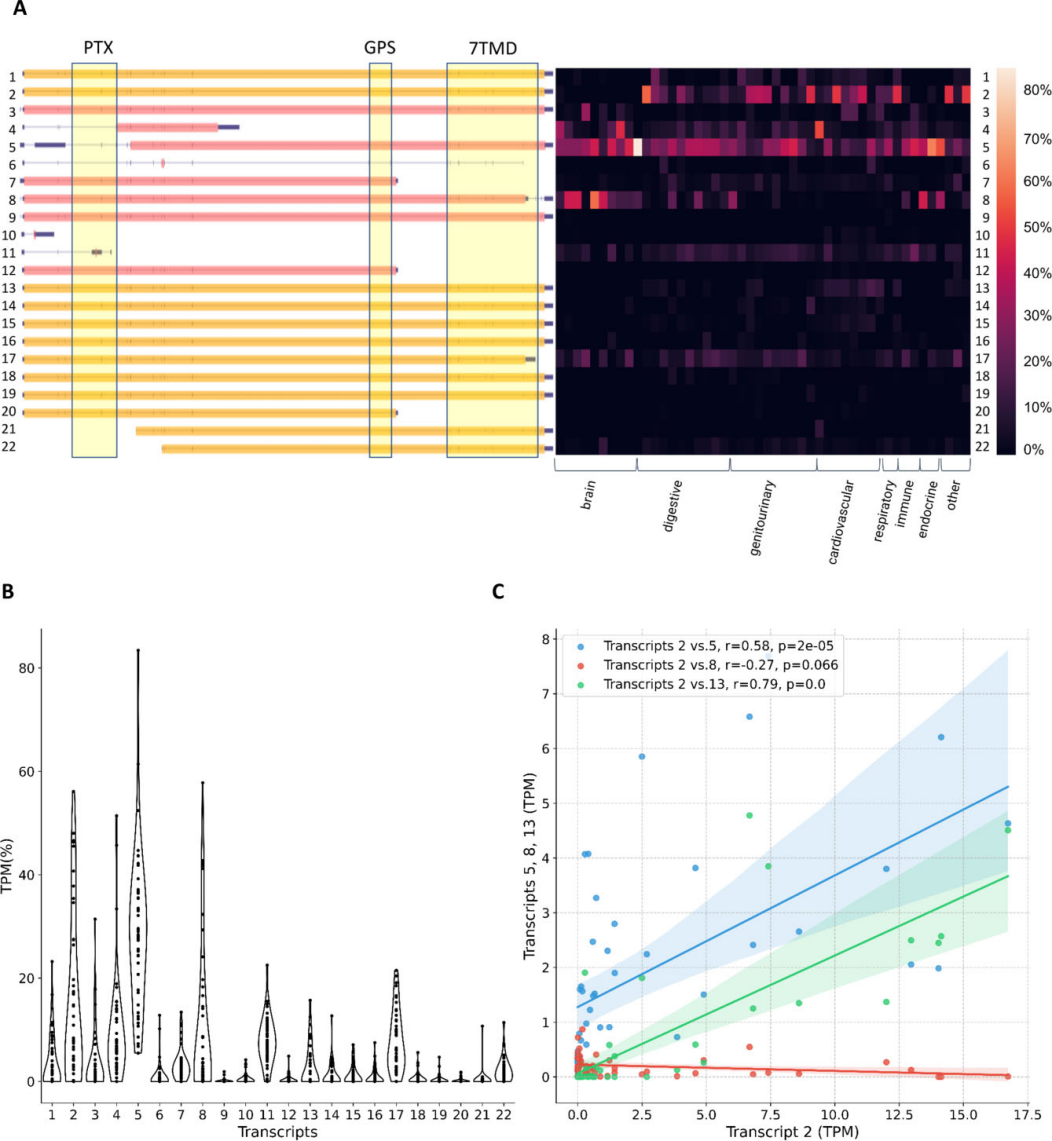
Tissue-specific transcript variants of *ADGRD1/GPR133*. (**A**) Transcript variants and ORFs of *ADGRD1/GPR133* are depicted (orange: already annotated, red: newly identified). Protein domains depicted as yellow boxes. Heatmap shows relative expression of each transcript variant in % of the total transcript variant count per each tissue (Supplementary Table S5). (**B**) Contribution of 22 transcripts to overall *ADGRD1* expression, each violin plot contains all 48 tissues. (**C**) Pearson correlation of absolute expression of transcript variants within *ADGRD1*, specifically transcript variants 2, 5, 8 and 13. The individual data points represent different tissues, 95% confidence intervals are shown. PTX, Pentraxin domain. GPS, G protein-coupled receptor proteolytic site. 7TMD, seven-transmembrane helix domain.

We did not only quantify tissue specificity of aGPCR expression, but also the contribution of the different transcript variants to the total aGPCR mRNA amount. Exemplarily, we present and discuss such an analysis for *ADGRD1/GPR133*. As shown in Figure 1, this aGPCR has 22 transcript variants in human tissues. *ADGRD1* transcript variants 5 and 8 (Figure 1A, ORF in red), which lack the PTX domain and parts of the 7TMD, respectively, are not annotated yet, but were the most abundant transcript variants in brain and several other tissues. A heat map of *ADGRD1* expression shows that the relative abundance of the individual transcript variants is tissue-specific and is not a result of proportional splicing. This would occur when e.g., two alternative transcript variants differ in their expression, however, maintain their ratio to each other. To verify the impression of tissue-specific splicing visualized in the heat map, we plotted the frequencies of various *ADGRD1* transcript variants (each bin contains all tissues, Figure 1B), which already shows unequal distributions of transcript variant frequency. In the case of pan-tissue (not tissue-specific) expression, rather normal distributions were expected. Furthermore, we analyzed all transcript variants with respect to their co-expression, where in the case of proportional splicing, a significant positive correlation would be expected. As displayed for the abundant *ADGRD1* transcript variants 2 and 8 (Figure 1A), there is no significant correlation indicating an independent tissue-specific transcription/splicing (Figure 1C). However, transcript variants 2 and 5, as well as transcript variants 2 and 13 were positively correlated (*r* = 0.58 and *r* = 0.27, Figure 1C) suggesting some dependency during transcription and/or splicing.

Tissue-specific transcription and splicing is not unique to *ADGRD1* and was observed for many aGPCRs (Supplementary Table S5). Multiple promoters and complex exon-intron structures of aGPCR genes contributed to this transcript variability and this is in line with the specific functions of aGPCRs in specific tissues. Future experimental planning should consider that the annotated aGPCR transcript variants may not be the dominant transcript variant, and that assays (histological, mRNA probes, antibodies) and functional studies (e.g., knock-out mouse) should be adapted to the specific transcript variant repertoire in the tissue of interest.

### Structural repertoire of aGPCRs

**Figure 2.**
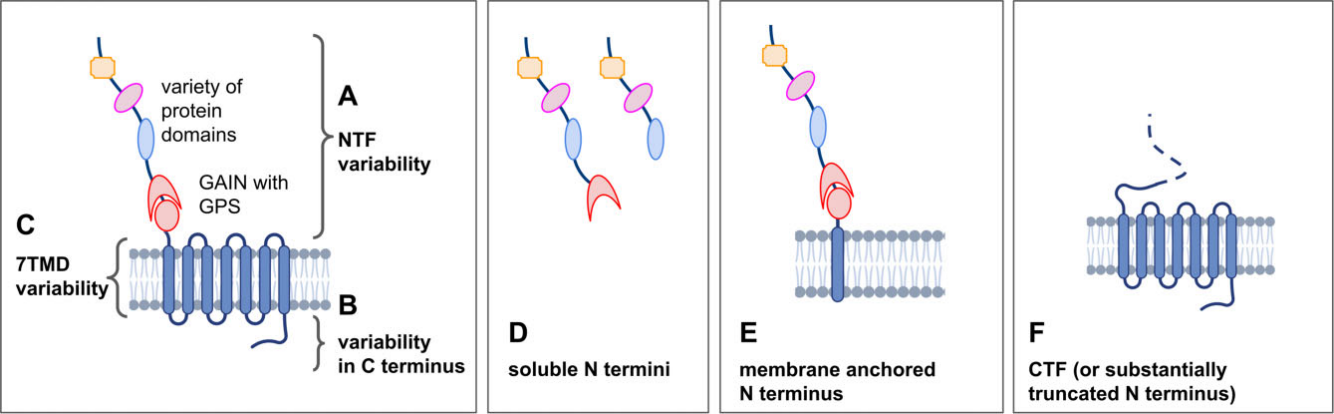
Structural variability of aGPCR isoforms resulting from different transcript variants. The structural variability of aGPCR isoforms are classified into isoforms with (**A**) NTF domain variability, (**B**) a variability in the C terminus, (**C**) variability in the 7TMD, (**D**) mRNA encoding for soluble N termini, (**E**) membrane-anchored N terminus and (**F**) CTF or substantially truncated N terminus. 7TMD, seven-transmembrane helix domain; CTF, C-terminal fragment; NTF, N-terminal fragment. The figure was created with BioRender.com.

The identified transcript variants varied in their exon composition, which also determined differences in the ORFs. Therefore, all transcript variants were screened for the longest ORF, and domains were annotated based on sequence homologies deposited in the Pfam database to evaluate the structural variability of resulting isoforms (Table 2). We categorized putative aGPCR isoforms as shown in Figure 2. About 88% of all aGPCRs showed sequence variability or even differences of domain composition in their N termini (Table 2, Figure 2A). This variability appears to provide a functional modularity mainly caused by alternative exon usage, exon skipping and intron retention during transcription and splicing. Similarly, 64% of aGPCRs showed variability in their C termini again due to alternative exon usage, alternative donor or acceptor splice sites and termination of transcription (Figure 2B). Less variability was observed in the 7TMD, with most variability occurring in the loops (Figure 2C). N-terminally truncated aGPCR transcripts and even CTF are derived from almost half of the aGPCR genes (Figure 2F). Most interestingly, many soluble N termini of different sizes are not derived by cleavage at the GPS, but rather result from premature stops due to alternative splicing and intron retention (e.g., *ADGRB3* in Table 2, Figure 2D). Furthermore, membrane-anchored N termini are frequently seen mainly because of truncations within the 7TMD (Figure 2E). This protein isoform variability derived from aGPCR genes implies multiple *cis* functions (G-protein coupling) of full-length receptors and also CTF. G protein-independent functions, where the N terminus alone mediates signaling to other cells or different interacting partners (*trans* function) (37,38), can be realized with soluble or membrane-anchored N termini (e.g. *ADGRA3*).

Of note, N-terminally truncated isoforms including CTFs are predicted proteins based on the longest ORF. It needs to be tested whether these ORFs are indeed translated. It is also reasonable to assume that translation starts at a different position determined by an optimal Kozak sequence or an internal re-entry site of translation (IRES). Sometimes there are multiple translational start points not always showing a preference for the first in-frame *AUG* (39). Furthermore, signal peptide sequences, which we screened for in N-terminally truncated protein sequences (17,18), were missing in many ORFs of identified transcript variants (Table 2). However, this does not always affect membrane insertion as previously tested, e.g., for mouse ADGRD1/Gpr133 (4). Most rhodopsin-like GPCRs, which typically have short N termini, lack a signal peptide at their N terminus, except for those with large N termini (e.g., glycoprotein hormone receptors).

**Figure 3.**
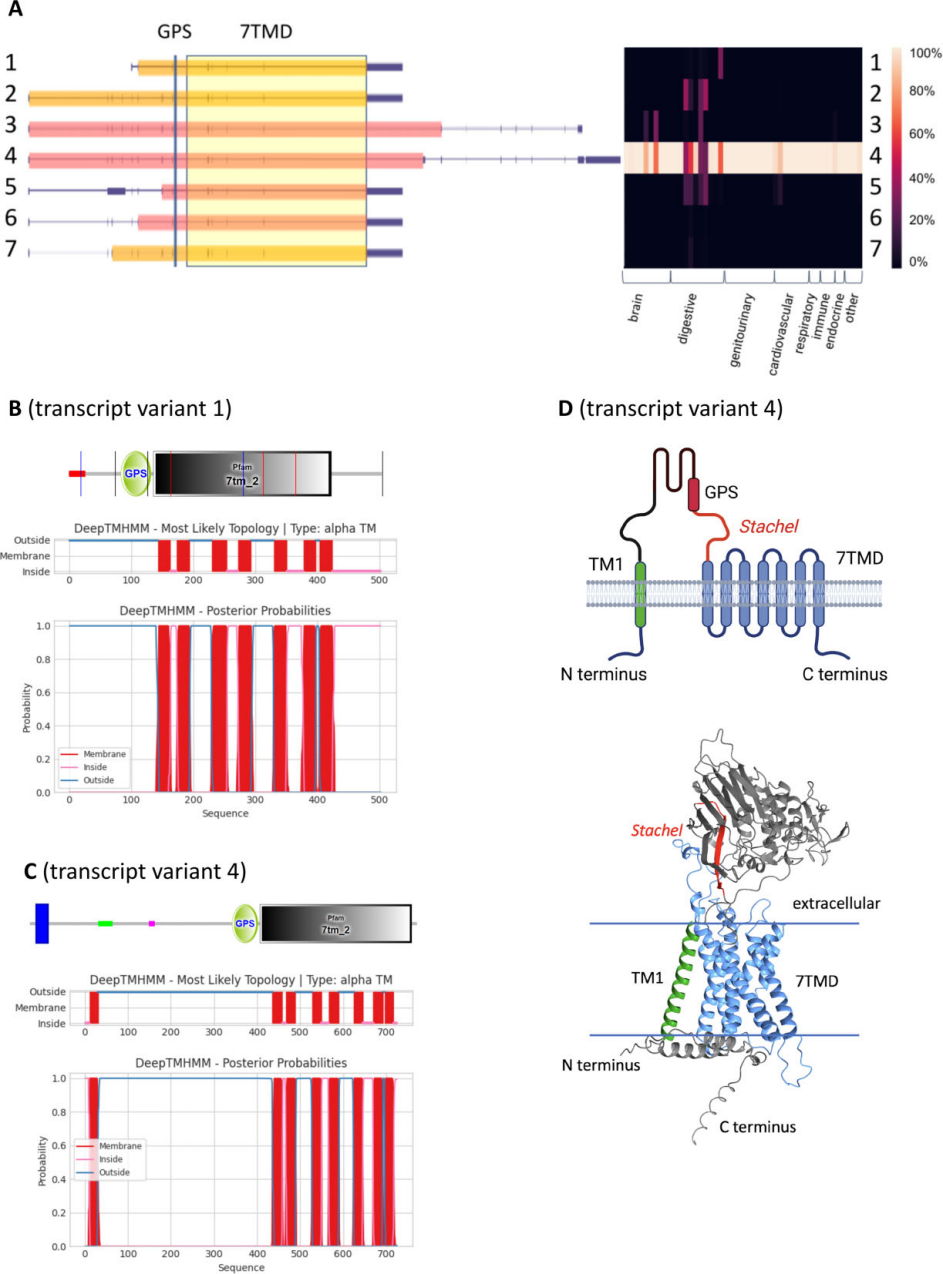
Predicted transmembrane helix topology of ADGRG7/GPR128. (**A**) *ADGRG7* has seven transcript variants, among which the novel transcript variant 4 (NSTRG.57932.4) showed highest expression in most tissues. The annotated transcript variants 1 and 2 (NM_001308362.1 and NM_032787.3) showed expression at significant levels in some tissues such as colon, small intestine, and liver. Heatmap shows relative expression of each transcript variant in % of the total transcript variant count per each tissue (Supplementary Table S5). Amino acid sequences of human ADGRG7 analyzed with SMART and DeepTMHMM predicted the GPS and 7TMD in all transcript variants and an additional transmembrane helix in transcript variants 2, 3 and 4 at the very N terminus. A signal peptide was only predicted in transcript variant 1 (**B**, red) and 6, however, not in transcript variant 4 (**C**). (**D**) In a hypothetical model of the ADGRG7 protein derived from transcript variant 4, the N- and C termini were located intracellularly. The three-dimensional model of the isoform derived from the long *ADGRG7* transcript variant (Q96K78) generated by AlphaFold (AF-Q96K78-F1-model_v4.pdb) similarly revealed eight transmembrane helices, with the N terminus located intracellularly. The figure was partly created with BioRender.com.

Strikingly, domain prediction revealed a transmembrane helix at the very N terminus of some human aGPCRs (ADGRA3, ADGRB1, ADGRG6, ADGRG7, ADGRL1) (19) additionally to the 7TMD. In case of ADGRA3, ADGRB1, ADGRG6 and ADGRL1, but not in case of ADGRG7 the predicted first transmembrane helix overlaps with the predicted signal peptide sequence. In fact, ADGRG7 presents with very unique features. As shown in Figure 3A, the human *ADGRG7* has 7 transcript variants where one (transcript variant 4) of the three long transcript variants (2–4) is the most abundant transcript variant. According to the domain prediction tools SMART and DeepTMHMM, all ADGRG7 protein isoforms contain the GPS and 7TMD. In contrast to the shorter transcript variant (e.g., transcript variant 1, NM_001308362.1, Figure 3B), all predictions for long transcript variants of the human *ADGRG7* (e.g., transcript variant 4, NSTRG.57932.4) lacked an N-terminal signal peptide, but included an N-terminal transmembrane helix additionally to the 7TMD (Figure 3C). Furthermore, we validated the newly identified TM with two other tools (MEMSAT3 (http://bioinf.cs.ucl.ac.uk/psipred); Phobius https://phobius.sbc.su.se/) meeting the standards of the annotation pipeline of UniProt (https://www.uniprot.org/help/transmem) to discriminate between TMs and signal peptides. The 295-amino acid-residue shorter transcript variant 1 (NM_001308362.1) contained an N-terminal signal peptide sequence and was generated by an intronic promoter (Figure 3A, B).

Essentially identical results were obtained with the mouse ortholog (NP_766413.2) and the amino acid alignment of the predicted N-terminal transmembrane helices showed high evolutionary conservation of hydrophobic amino acid residues among vertebrate orthologs (Supplementary Figure S4). The newly identified TM is present in sharks and very basal ray-finned fishes but not in other ray-finned fishes where the receptor sequence usually starts with a signal peptide sequence (Supplementary Figure S5A). Plotting the TM probability scores (implemented in DeepTMHMM) of the newly identified TM of selected vertebrate ADGRG7 sequences revealed a very similar pattern and length as found for the TM4 of the 7TMD (Supplementary Figure S5B). The TM4 was chosen as control because the N- and C terminus of TM4 is similarly oriented extra- and intracellularly, respectively, as the newly identified TM.

Consistently, three-dimensional structure prediction with AlphaFold (21) of the human and >30 vertebrate *ADGRG7* orthologs revealed the presence of this additional N-terminal transmembrane α-helix (Figure 3D). This generates an unusual structure previously unknown for aGPCRs with the N- and C terminus located intracellularly. The predicted alignment error (PAE), which is useful for assessing interdomain accuracy, is one parameter of the AlphaFold model quality. Here, the values and their pattern within the new TM is very similar to those for helices of the 7TMD (Supplementary Figure S6). Moreover, PAE values reflecting the orientation and distance of the new TM to the 7TMD suggest that they form a transmembrane bundle consisting of eight transmembrane helices.

Autoproteolysis at the GPS can split the membrane protein into a membrane-anchored NTF and a GPCR-like CTF. This unique feature of aGPCR may be relevant for the activation mechanism of this receptor class. Several aGPCRs have been identified as mechanosensors, and a separation of the NTF and CTF by mechanical forces after autoproteolytic cleavage at the GPS may lead to the exposure of the *Stachel* sequence from the GAIN domain for 7TMD binding and activation (reviewed in (40–42)).

**Figure 4.**
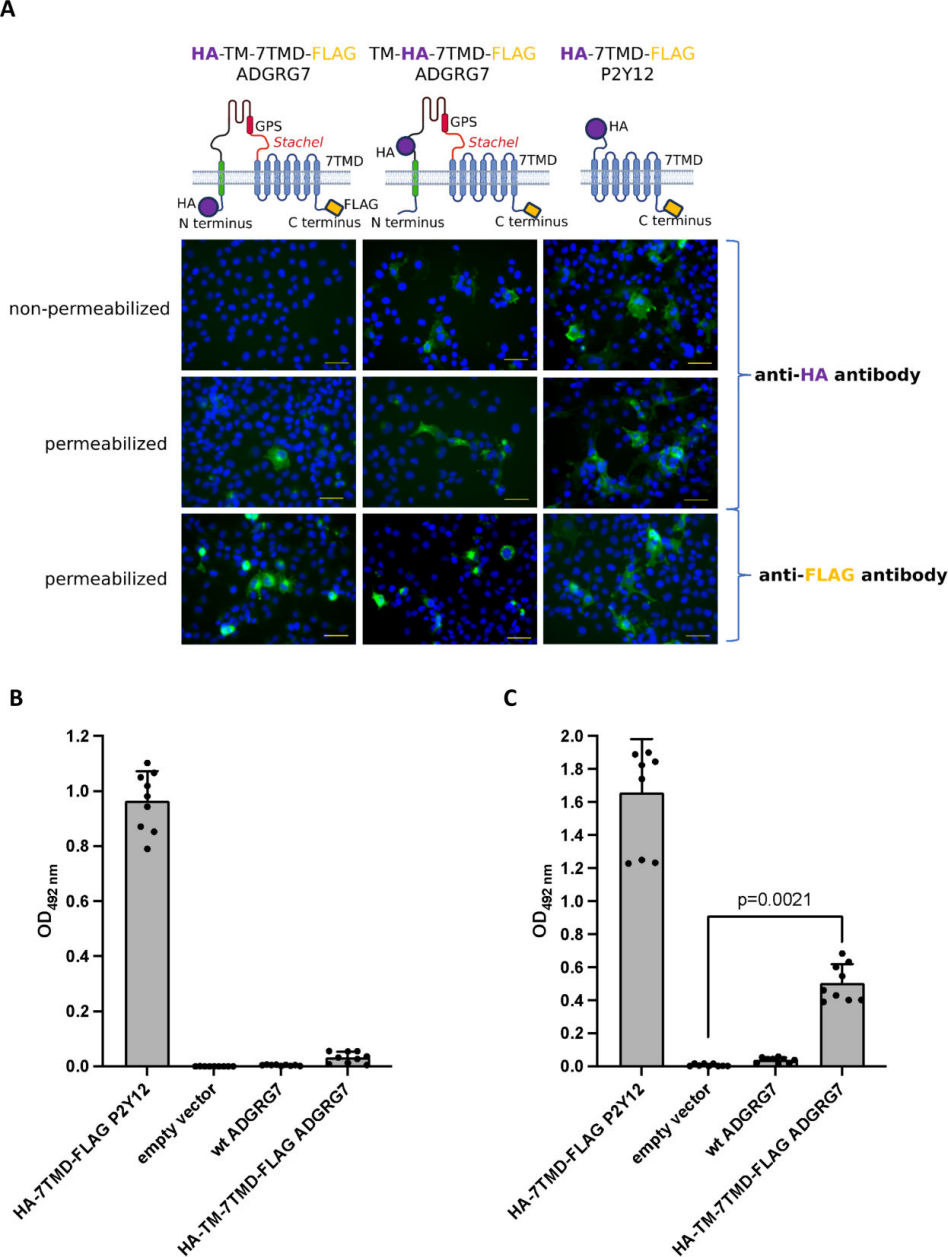
The N- and C terminus of ADGRG7/GPR128 is located intracellularly. COS-7 cells were transiently transfected with the double-tagged mouse ADGRG7/GPR128 and investigated in immunofluorescence (**A**) and ELISA (B, C). For immunofluorescence transfected cells were permeabilized and incubated with an antibody against the HA tag and an antibody against the C-terminal FLAG tag (as indicated for the constructs). The monoclonal antibodies were stained with a secondary goat anti-mouse IgG AlexaFluor488 nm-conjugated antibody (green). DAPI (blue) was used to visualize the nucleus and confocal images were taken with the BZ-X810 Fluorescence Microscope, Keyence with a 20x objective. Representative images are shown. The bar indicates 25 μm. Cell surface (**B**) and sandwich ELISA (**C**) tests were performed to determine the expression levels at the non-permeabilized plasma membrane and the solubilized full-length receptor, respectively. Shown is the mean ± SD of three independent assays performed in triplicate. The statistical significance was tested with a Student's *t*-test over the mean of biological replicates (*n* = 3). The figure was partly created with BioRender.com.

To experimentally test whether the predicted topology with a cytosolic localization of both the N- and C terminus is true, we generated an N-terminally HA-tagged (N-terminally of the new TM) and C-terminally FLAG-tagged ADGRG7/GPR128 (HA-TM-7TMD-FLAG) and expressed it in COS-7 cells. First, we studied the localization of the N-terminal HA tag in immunofluorescence microscopy. In case the N terminus of ADGRG7/GPR128 would be located extracellularly, non-permeabilized cells would show a specific fluorescence at the plasma membrane. Because no specific signal was detected in non-permeabilized cells, we permeabilized the HA-TM-7TMD-FLAG-transfected cells with Triton-X100 and found intensive staining of cellular membranes (Figure 4A). This indicates an intracellular localization of the N terminus of ADGRG7/GPR128. For control purposes of the anticipated topology, we introduced the HA tag C-terminally of the new TM (TM-HA-7TMD-FLAG) and found HA-tag specific signals already in non-permeabilized cells (Figure 4A) which were similar to the staining of a classical HA-tagged rhodopsin-like GPCR, the ADP receptor P2Y12 (HA-7TMD-FLAG P2Y12). The presence of all full-length constructs was verified with an antibody against the FLAG tag inserted at the very C terminus and permeabilized cells (Figure 4A).

To quantitatively validate the immunofluorescence findings, we performed cell surface ELISA. The N-terminally HA-tagged positive control (HA-7TMD-FLAG P2Y12) (43) showed a significant cell surface expression compared to the negative controls (empty vector, non-tagged ADGRG7) (Figure 4B). As expected from the immunofluorescence studies, the HA/FLAG-double-tagged ADGRG7/GPR128 (HA-TM-7TMD-FLAG) gave no signal above the negative control indicating that the N terminus is not located at the outside of the plasma membrane. To assure that the full-length ADGRG7/GPR128 is indeed translated, we applied a sandwich ELISA to detect HA/FLAG-double-tagged receptor protein from cell lysates. As shown in Figure 4C, the ELISA signal of HA-TM-7TMD-FLAG ADGRG7-transfected cells was significantly higher compared to the negative control. The lower signal in the sandwich ELISA is most probably due to a lower transfection efficiency of the much bigger ADGRG7/GPR128 plasmid and protein compared to the P2Y12 (802 AA versus 359 AA with epitope tags) and potentially because of partial cleavage at the GPS and NTF/CTF dissociation.

To our best knowledge, ADGRG7/GPR128 is the first GPCR with more than seven transmembrane helices. Previous annotations of GPR89A and GPR155 as ‘GPCR’, containing more than seven transmembrane helices, were later proven wrong showing functions as voltage-dependent anion channel (44) and lysosomal multi domain transmembrane protein involved in cholesterol metabolism (45), respectively.

In summary, transcript variant analysis revealed a comprehensive portfolio of potential protein isoforms derived from one gene. In case of aGPCRs, analysis of the entire receptor class exposed a common pattern of frequent protein isoforms with different functions (soluble N termini, anchored N termini, CTF).

### Clinical implication

Premature stop codons and frame-shifting mutations are often considered inactivating for the gene function and causative for phenotypes or genetic diseases. Given multiple transcript variants of one gene, mutations and polymorphisms may affect only specific transcript variants. To analyze their potential effects, we included a feature in the application that shows a given genomic position on the various transcript variants of a gene.

**Figure 5.**
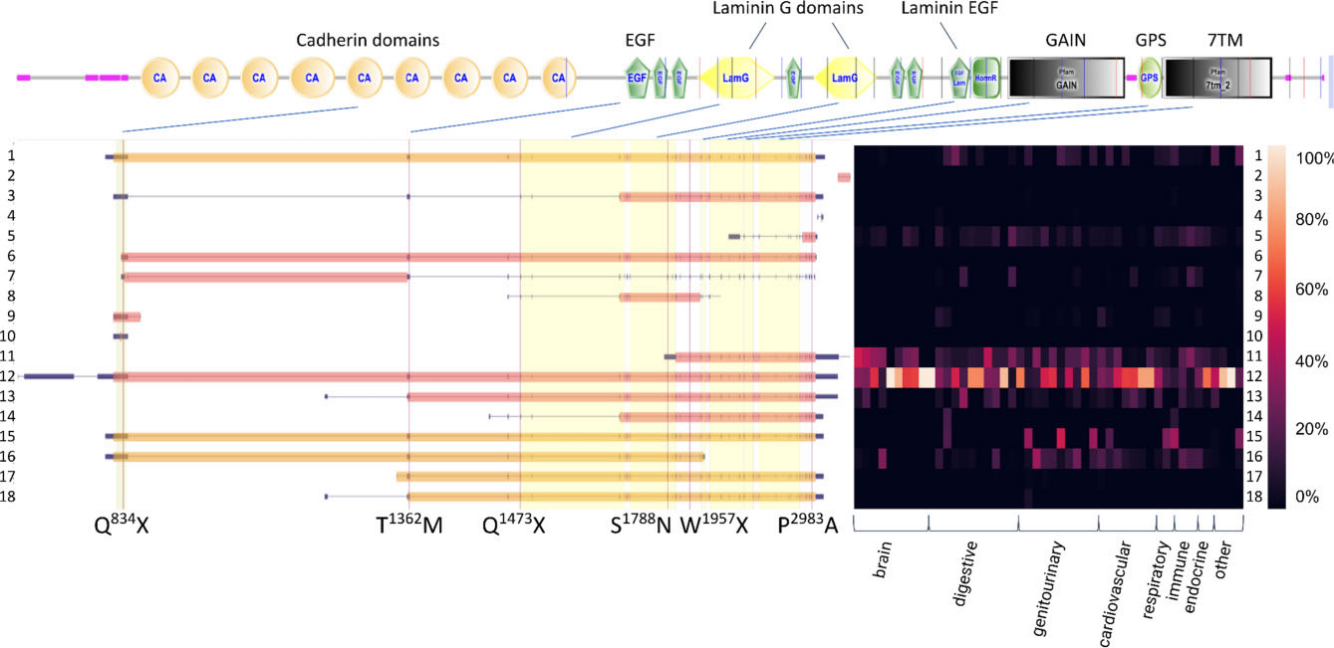
Projection of *ADGRC1/CELSR1* mutations found in patients with neural tube defects. (Likely) pathogenic mutations from the Human Gene Mutation Database (HGMD®, https://www.hgmd.cf.ac.uk/ac/index.php) were projected onto the transcript variants. Depending on the position of the mutations, a subset of transcript variants may remain unaffected. Domain structure is given on top (generated by the SMART, see Materials and methods). Heatmap shows relative expression of each transcript variant in % of the total transcript variant count per each tissue (Supplementary Table S5).

Functionally relevant mutations in GPCR genes, including aGPCR genes, are responsible for about 66 inherited monogenic diseases in humans (46). For example, mutations in the *ADGRC1*/*CELSR1* genes are associated with developmental disorders such as neural tube defect, spina bifida, craniorachischisis, and congenital heart defects (47–49). More than two dozen mutations are described in the *ADGRC1*/*CELSR1* gene, which are claimed to be causative for these developmental defects (46). ADGRC1 is a large membrane protein with multiple transcript variants and protein isoforms (Tables 1 and 2) composed of up to 3000 amino acid residues. As shown in Figure 5, pathogenic mutations can affect all parts of the receptor protein. However, depending on the genomic position, not all transcript variants contained all pathogenic mutations. For example, transcript variants 11 and 12 were the most abundant transcript variants, and differed significantly in their predicted isoform structure. While transcript variant 12 represented the full-length receptor containing all domains, transcript variant 11 was generated presumably using an internal promoter and should contain only the GAIN and 7TM domains. As a consequence, upstream mutations (e.g., Q^834^X, T^1362^M, Q^1473^X, S^1788^N) would not affect transcript variant 11, and only W^1957^X or P^2983^A would do so. Hence, this could lead to partial or variable phenotypes depending on which exon and, therefore, which transcript variants are altered by the mutation. Currently, the number of patients is too low to correlate different mutations with features of the phenotypic spectrum. Nevertheless, some genomic variants can already be evaluated with respect to their clinical relevance. It should be noted that the transcript variants analyzed here originate primarily from the analysis of tissues from adult individuals (exception: fetal brain tissue). Therefore, subsequent analyses taking into account different developmental stages may uncover even more transcript variants. The C terminus of ADGRC1 is variable in length and sequence, indicating that mutations within this region will probably have a lower functional impact. P^2983^A has been associated with craniorachischisis and functional assays showed that this mutant aGPCR seems to be retained in the cellular interior (47). However, the position P^2983^ is located in the variable C terminus of this aGPCR, and shows low evolutionary conservation (e.g., the corresponding position is a leucine and an alanine in several bat and guinea pig *ADGRC1* orthologs, respectively). Also, the variant allele is frequent in the gnomAD population, including homozygote humans without obvious phenotypes, thus, pathogenicity of the described mutant needs to be questioned. Therefore, analyzing transcript variant variability can support the evaluation of potentially disease-causing mutations and may explain phenotype variation across tissues and individuals. This is of great importance not only for aGPCRs, but also for other protein classes in which a large number of disease-causing mutations have been described.

In the future, human genetic approaches may benefit from the inclusion of new exons that were not previously part of diagnostic exome sequencing or part of genomic variant assessment databases such as gnomAD. Using *ADGRC1* as an example, we identified 30 out of 72 exons that included regions which were not covered by currently used exome sequencing approaches, such as Twist Bioscience. Some exons were also not included in the annotation currently used in gnomAD (Supplementary Table S7). Some of these exons, such as exon 9 from the newly identified and expressed transcript variant NSTRG.54985.12, are even part of the predicted coding region. As a result, genomic variants that affect these newly identified regions will be missed by standard diagnostic approaches.

## Conclusion

Our analysis demonstrates that the repertoire of aGPCR transcript variants and possibly also protein isoforms is higher than the number of aGPCR genes. This structural variability does not only give rise to variable domain composition of aGPCRs, but most probably also to unusual transmembrane helix composition and membrane-anchoring of the NTF as in case of ADGRG7. Moreover, the tissue-specific repertoire of aGPCR transcript variants could gain relevance for the interpretation of disease-causing mutations. Hence, extending beyond the structure of aGPCRs, our study has shown that analyses of tissue-specific transcriptomes are feasible and can increase our understanding of the transcript variant repertoire of human genes. Our presented webtool provides a starting point for the analysis of tissue-specific transcript variants for other gene families as well.

## Glossary

**C terminus** The section of the protein from the C-terminal amino acid residue to the last transmembrane helix. In aGPCRs, the C terminus is normally known to enable interaction with intracellular signaling effectors.

**CTF** Membrane-spanning C-terminal fragment of aGPCRs generated by autoproteolytic cleavage of the N terminus at the G protein-coupled receptor proteolysis site (GPS) which is part of the GAIN domain.

**Chimeric Transcript** A transcript that incorporates sequence from two independent genes. It can occur at the genomic level as a result of translocation, interstitial deletion, or chromosomal inversion but also at the transcription level due to RNA polymerase read-through or exon skipping.

**Internal promoter** On the basis of the DNA sequence coding for the full-length protein, the internal promoter is defined as the nucleotide sequence to which the RNA polymerase binds and initiates transcription, which is located within this DNA sequence. This results in shorter mRNA transcripts and eventually shorter protein sequence.

**N terminus** The section of the protein from the very N-terminal amino acid residue to the first transmembrane helix. In aGPCRs, the N terminus is normally known to receive extracellular signals, e.g., ligand binding or mechanical forces.

**NTF** Extracellular N-terminal fragment of aGPCRs generated by the autoproteolytic cleavage of the N terminus at the G protein-coupled receptor proteolysis site (GPS) which is part of the GAIN domain.

**Protein isoforms** A variant form of a protein, which may be encoded by different genes, e.g., the isoenzymes of lactate dehydrogenase, or by a single gene whose transcript is alternatively spliced, according to the NCBI terminology.

**Pseudogene** Presumably nonfunctional sequences of genomic DNA originally derived from a functional gene.

**Transcript** RNA molecule transcribed from DNA, which encodes proteins (mRNA) or is non-protein-coding (tRNA, rRNA etc.). Transcripts may vary with respect to their start and end coordinates, exon usage and RNA sequence. Relative transcript abundance can be measured in transcripts per million (TPM).

**Transcript variants** Different transcripts (mRNA transcripts) derived from the same gene, according to NCBI terminology.

## Supplementary Material

gkae145_Supplemental_Files

## Data Availability

The datasets used for this study are available in the Gene Expression Omnibus (GEO) repository (https://www.ncbi.nlm.nih.gov/geo/) under accession numbers GSE173955, GSE182321, GSE101521, GSE174478, GSE217427, GSE165303 (SRP302848), GSE138734 (SRP225193) and under PRJEB23709 at the European Nucleotide Archive (https://www.ebi.ac.uk/ena/browser/). *Splice-O-Mat* is a comprehensive online database available at https://tools.hornlab.org/Splice-O-Mat/. Code for the featured application and for conducted analyses are available at https://github.com/chrissikath/Splice-O-Mat and at https://zenodo.org/doi/10.5281/zenodo.8409466.
