# Supplementary material for: The repertoire and structure of adhesion GPCR transcript variants assembled from publicly available deep-sequenced human samples
Source: Nucleic Acids Res. 2024 Feb 29;52(7):3823–36. doi: 10.1093/nar/gkae145 (PMC11039983; doi:10.1093/nar/gkae145)
Supplement: gkae145_Supplemental_Files [file gkae145_supplemental_files.zip › Revised_Kuhn_et_al_Transcript_variants_of aGPCRs _20230420_Suppl.docx]

**Supplementary Information**

**for**

**The repertoire and structure of adhesion GPCR transcript variants assembled from publicly available deep-sequenced human samples**

**Authors**

Christina Katharina Kuhn^1^, Udo Stenzel^1^, Sandra Berndt^1^, Ines Liebscher^1^, Torsten Schöneberg^1,2*^, Susanne Horn^1,3*^

**Affiliations**

^1^ Rudolf Schönheimer Institute of Biochemistry, Medicinal Faculty, University of Leipzig, 04103 Leipzig, Germany

^2^ Department of Biochemistry, School of Medicine, University of Global Health Equity (UGHE), PO Box 6955, Kigali, Rwanda

^3^ Institute of Translational Genomics, Helmholtz Zentrum München - German Research Center for Environmental Health, 85764 Neuherberg, Germany

*Corresponding authors

Susanne Horn

Tel: +49 (0)341 97-22152

Fax: +49 (0)341 97-22159

E-Mail: susanne.horn@medizin.uni-leipzig.de

Torsten Schöneberg

Tel: +49 (0)341 97-22151

Fax: +49 (0)341 97-22159

E-Mail: torsten.[scho](mailto:Christina.Kuhn@medizin.uni-leipzig.de)eneberg@medizin.uni-leipzig.de

# **Supplementary Figures**


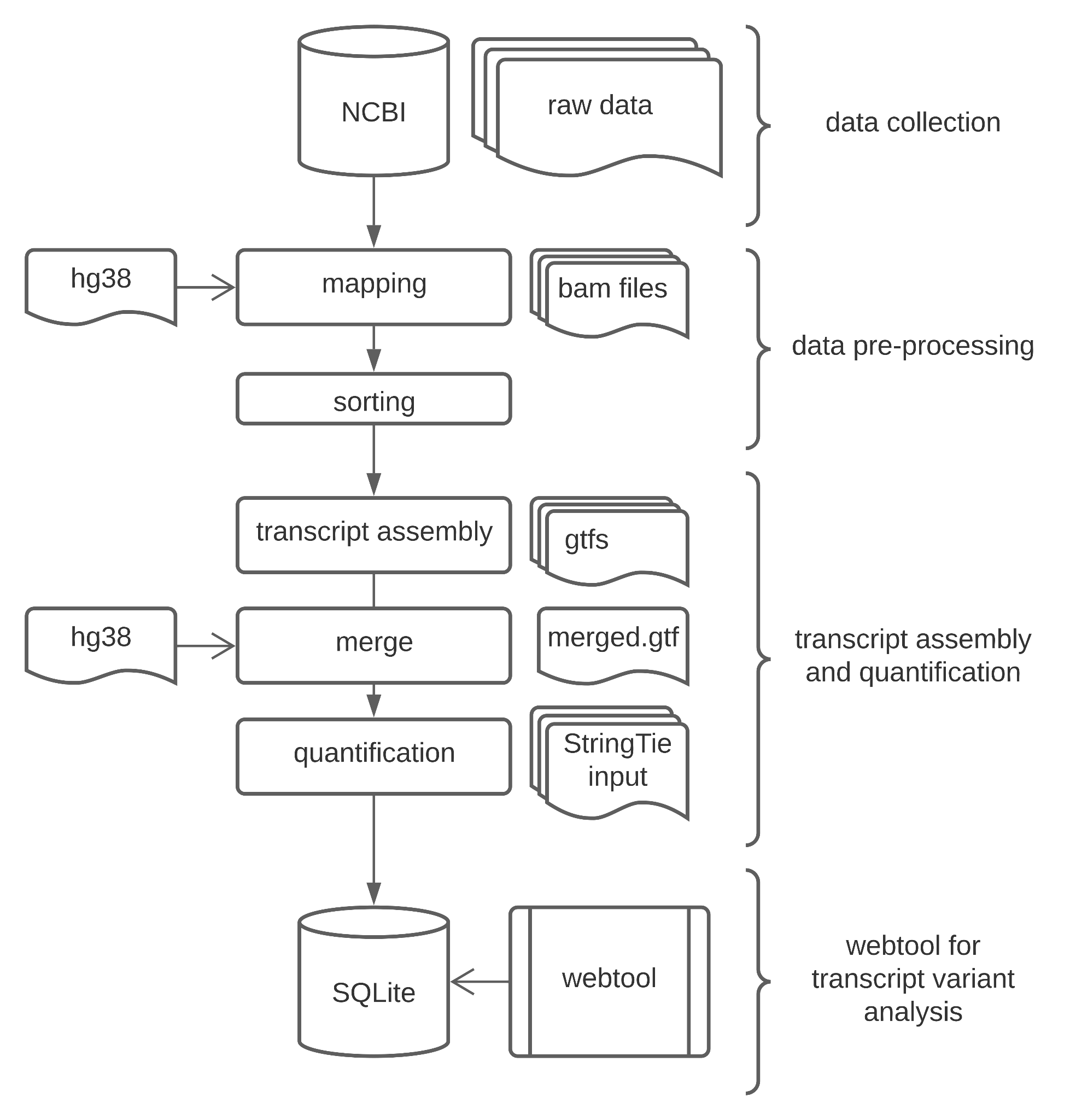


**Figure S1.** Workflow of the sequence analysis. Raw reads from the collected RNA-seq dataset were mapped to the human genome (GRCh38) using STAR version (version 2.7.6a) with default parameters [(1)](https://www.zotero.org/google-docs/?IUZiMb). After sorting, StringTie (version v2.1.3b) was used according to the manual [(2)](https://www.zotero.org/google-docs/?NV5iWV) for the assembly and transcript quantification of the mapped reads. To generate a global, unified set of transcript variants across RNA-Seq samples, StringTie merge mode was used providing the reference annotation. The resulting gtf files were fed into a SQLite database (version 3.39.3, <https://bitbucket.org/ustenzel/stringtiedb>). The webtool was implemented in python (version 3.7) using dash (version 2.6.2) and hosted on the green unicorn server (version 20.1.0) and is available at <https://tools.hornlab.org/Splice-O-Mat/>.

| A  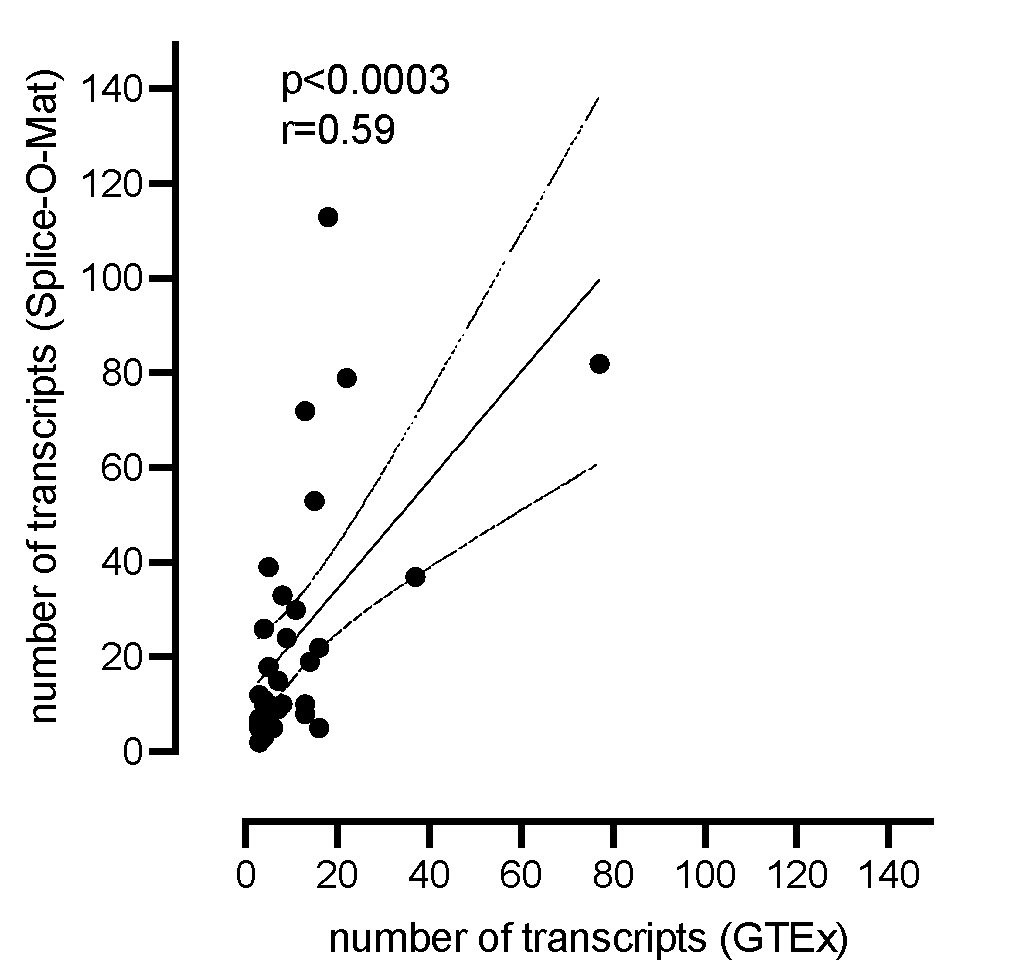 | B  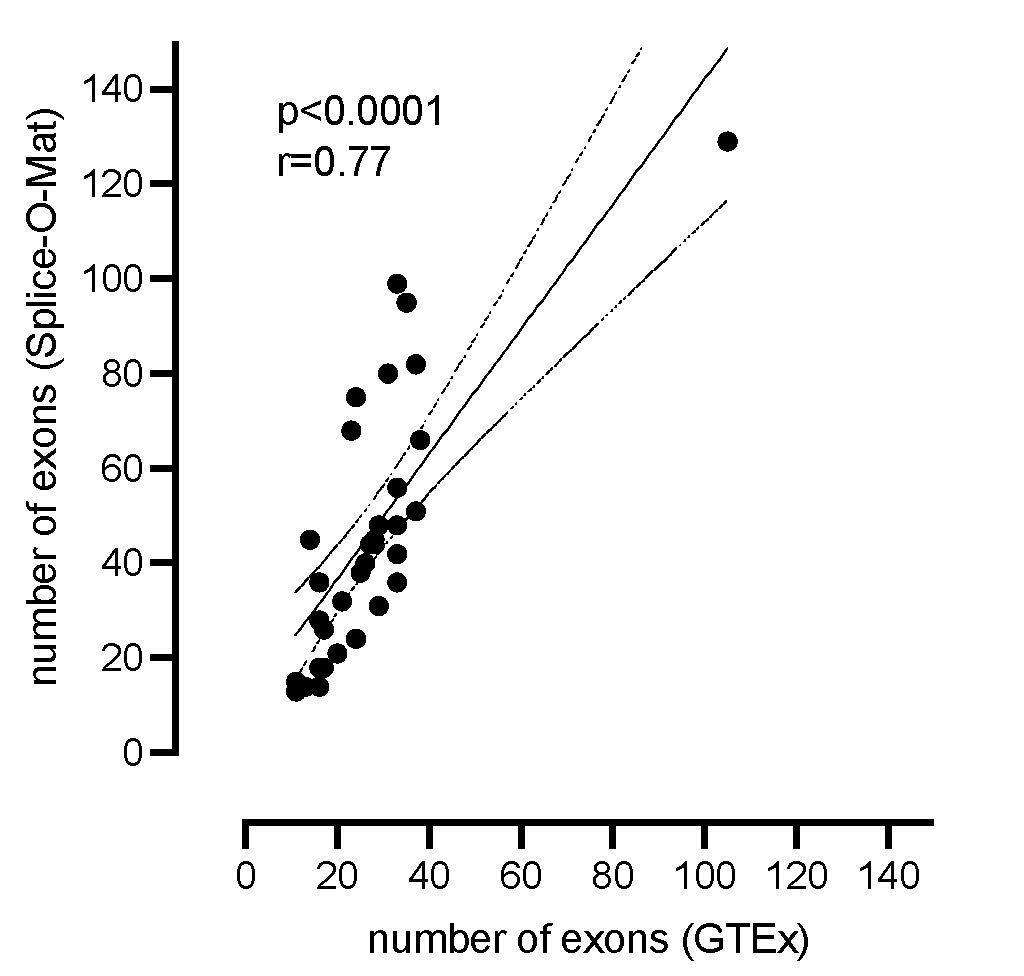 |
| --- | --- |

C


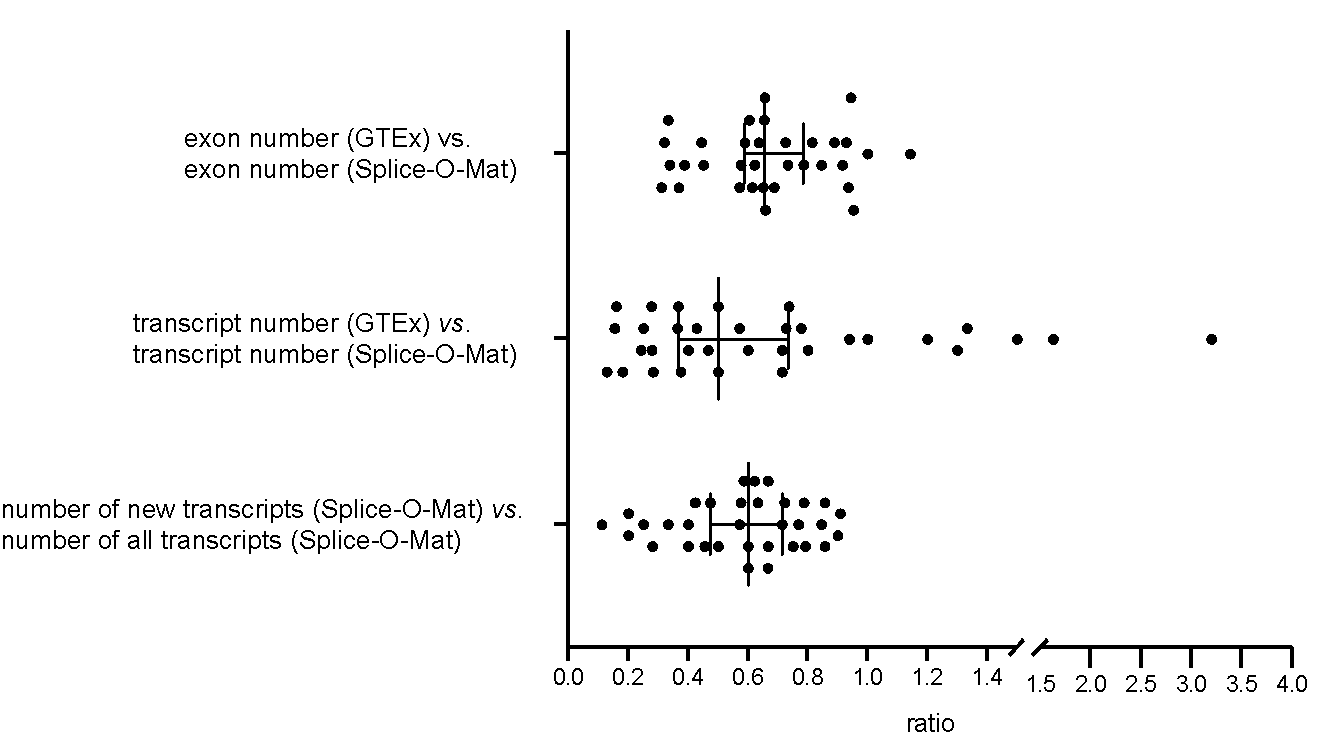


**Figure S2.** Comparison of identified transcript variants/exons with Splice-O-Mat with GTEx and NCBI. The number of transcript variants (**A**) and exons (**B**) from human aGPCR genes from GTEx and Splice-O-Mat webtools were plotted and correlated (Pearson). The respective linear regression curves with the 95% confidence interval are given. (**C**) Ratios of webtool-identified exon and transcript numbers (GTEx vs. Splice-O-Mat) and newly identified transcripts beyond already NCBI-annotated transcript variants (GRCh38) are shown as medians with the 95% confidence interval. GTEx v8 was used (GENOCDE 26 GRCh38.p10). N=33 in all GTEx-Splice-O-Mat comparisons. Full data can be found in [Supplementary Table S3.](https://docs.google.com/spreadsheets/d/1ftYsXVaJFIDxnbE3tFhp6_zOS4CJ7ekkmlymboi4_rg/edit#gid=251396919)

A


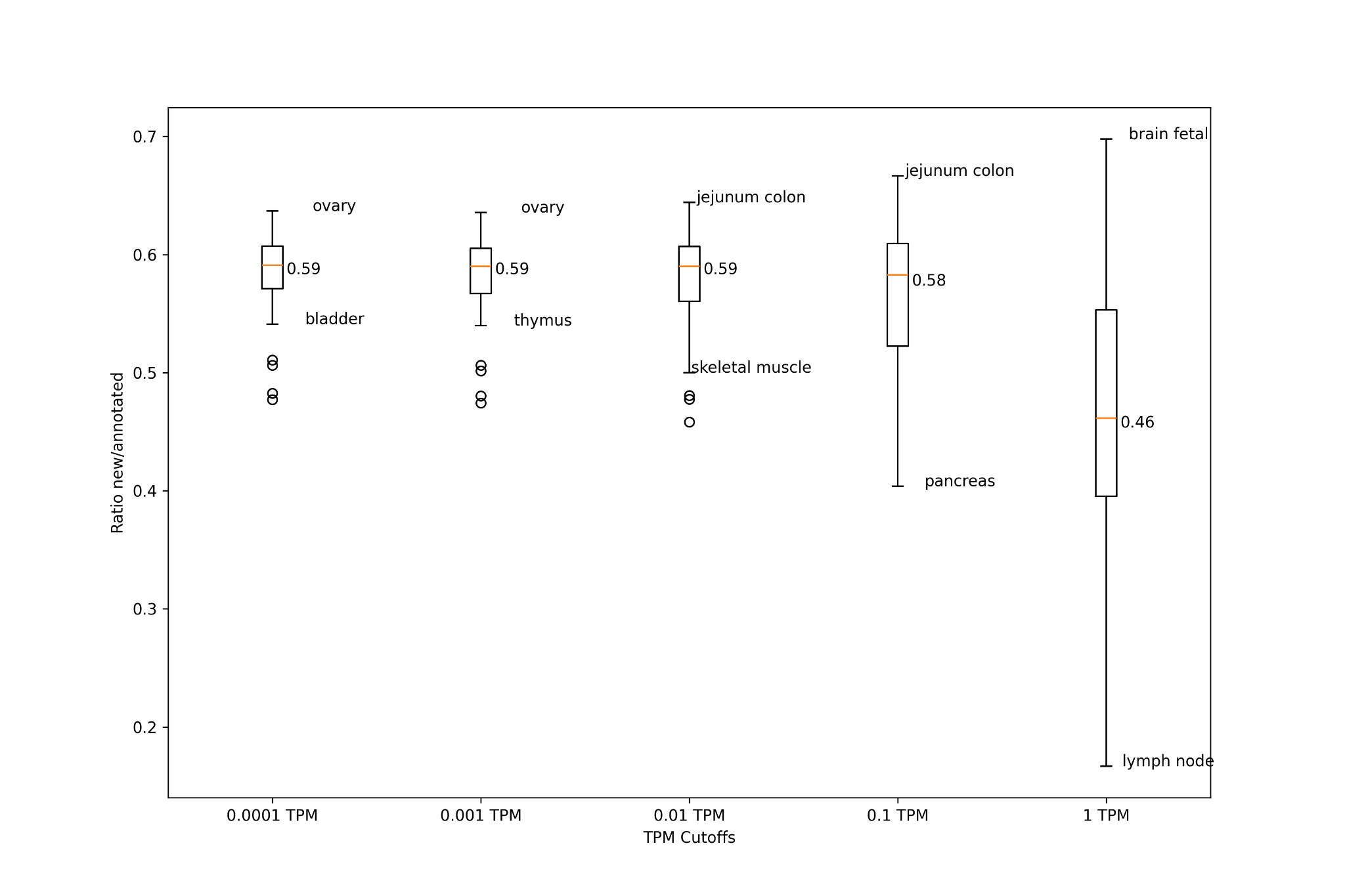


B

| 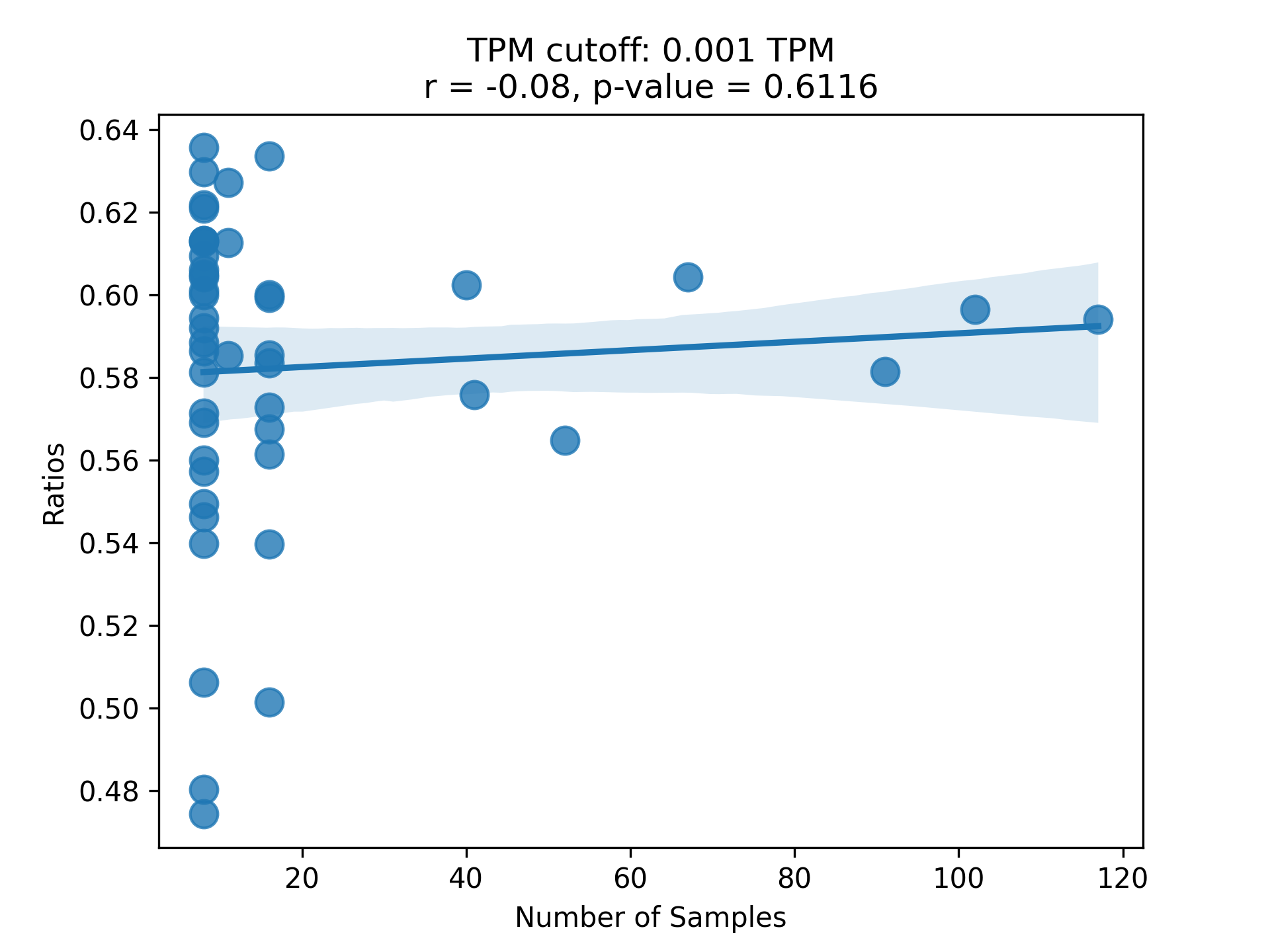 | 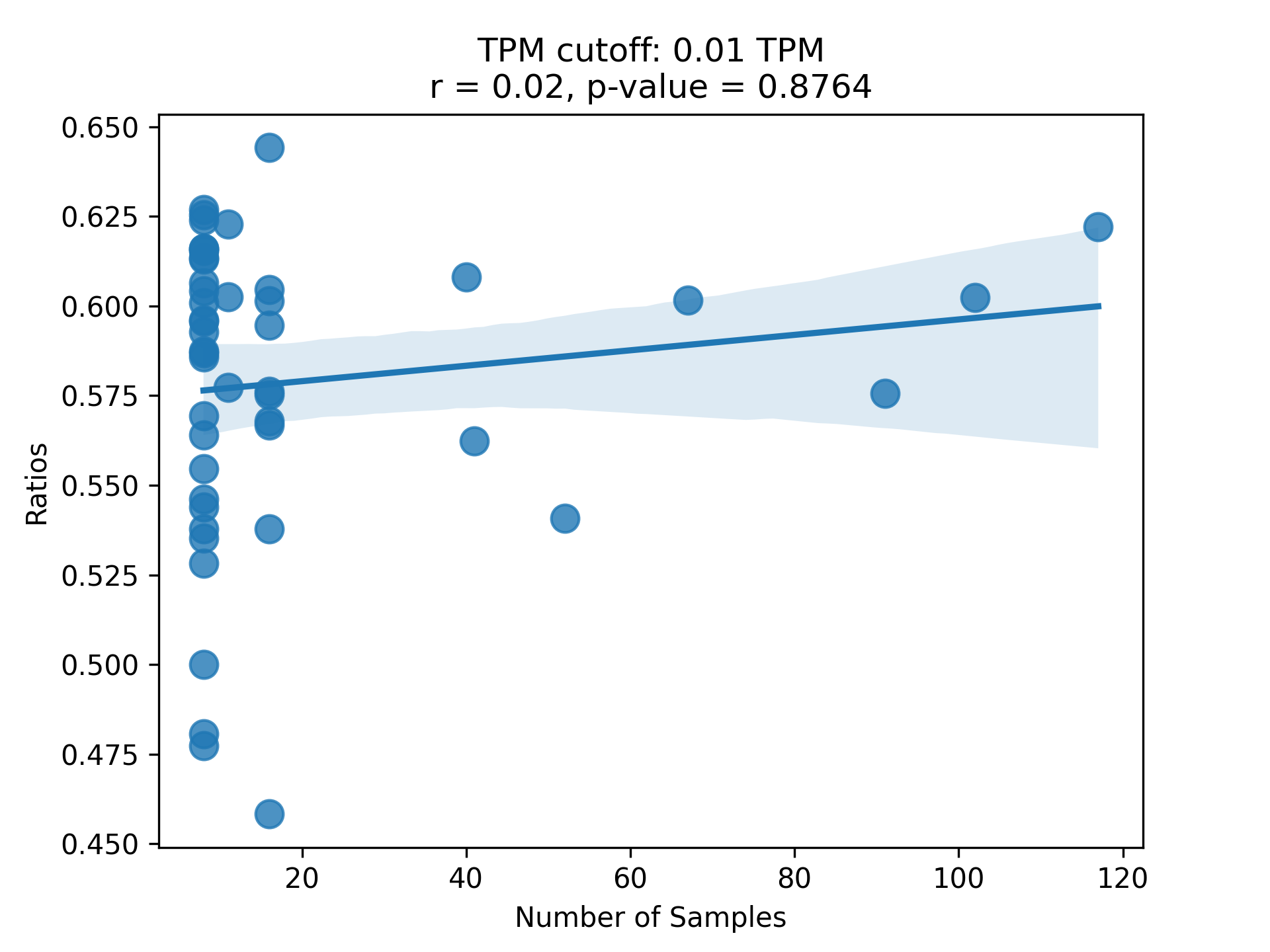 |
| --- | --- |
| 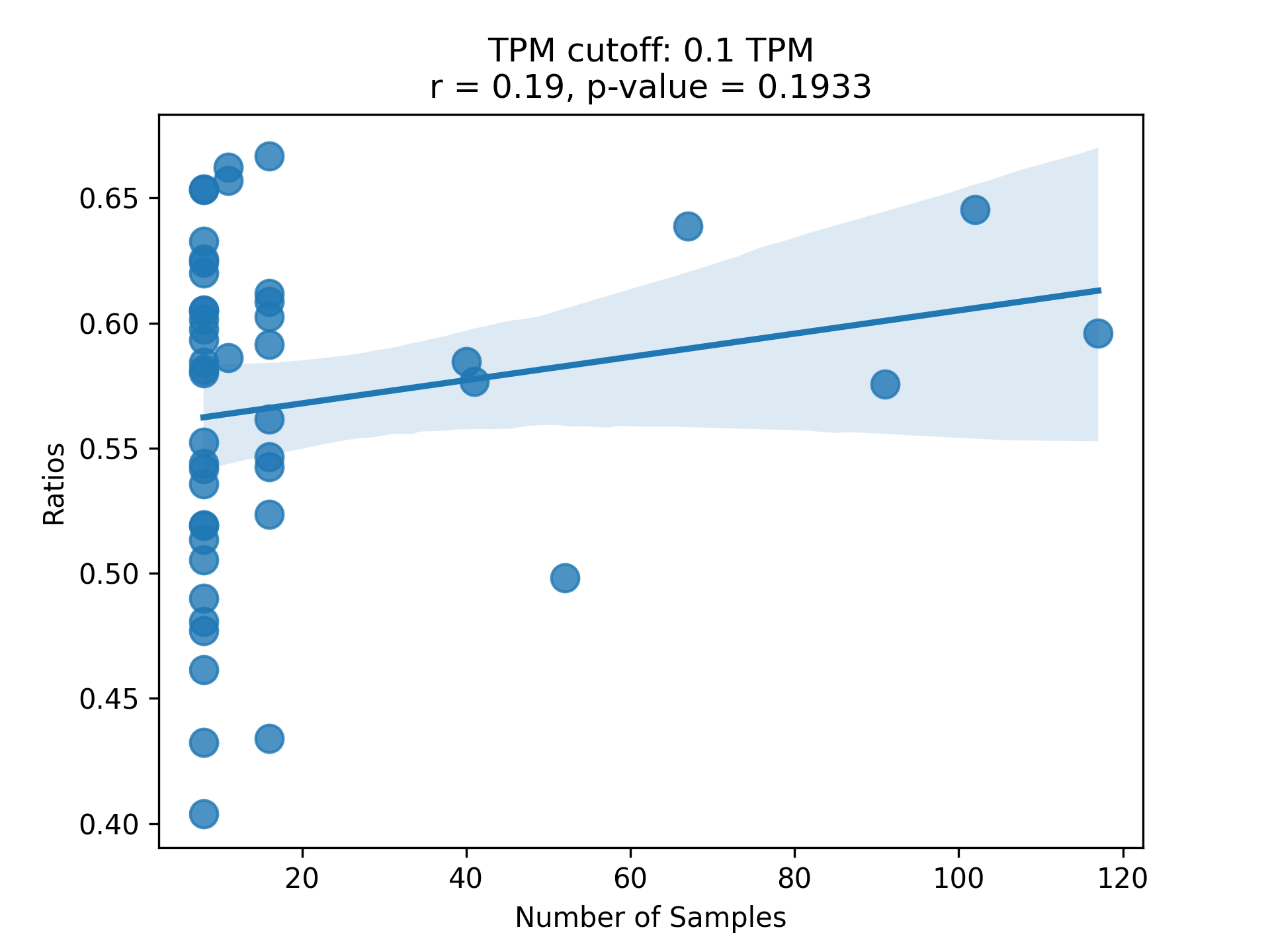 | 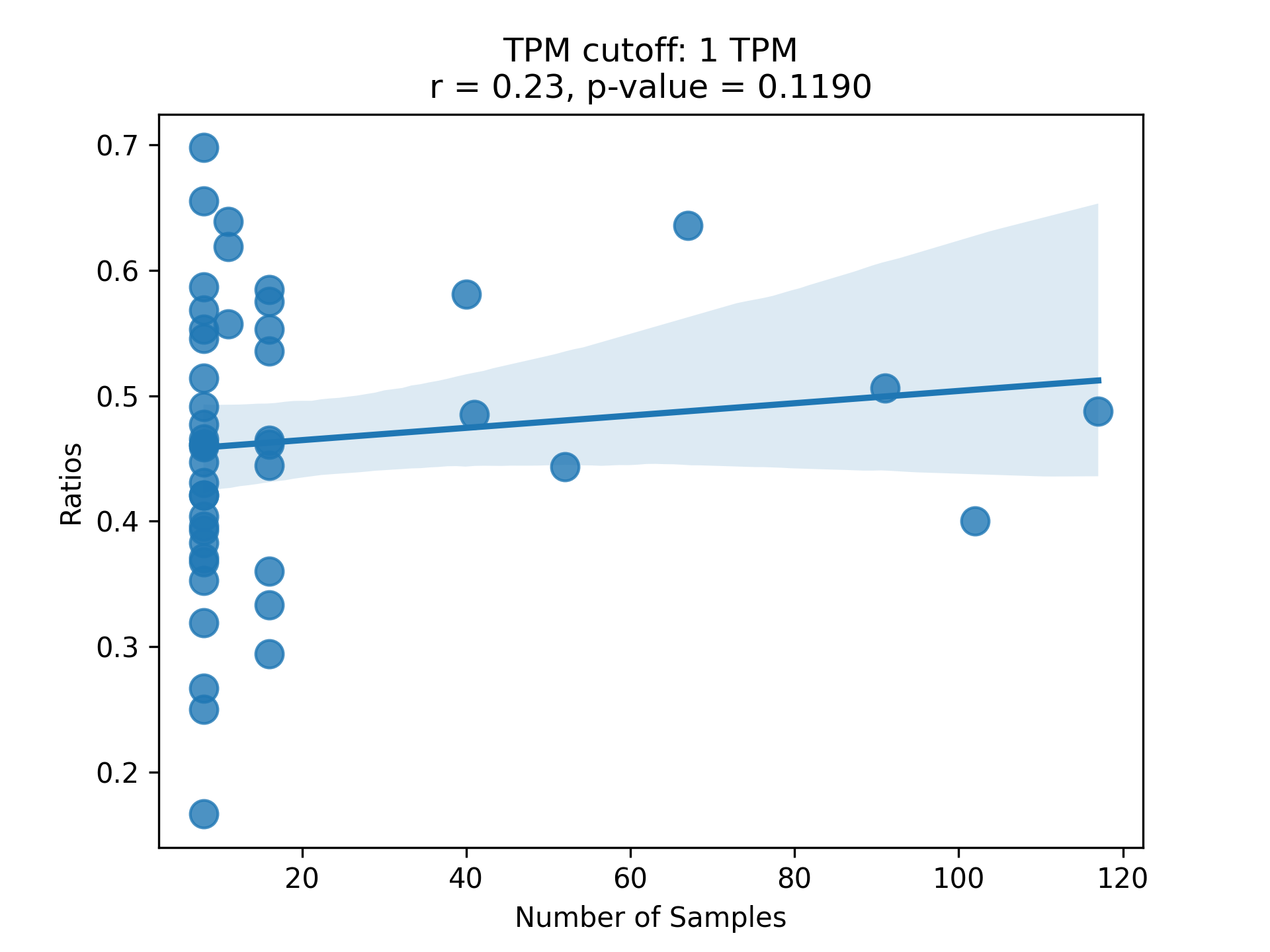 |

**Figure S3**. Ratio of newly identified transcript variants in relation to all identified transcript variants across varied expressed thresholds. Ratios were calculated by dividing the total count of newly identified transcripts per tissue by the count of transcript variants expressed above specific TPM thresholds, ranging from 0.0001 to 1 TPM. (**A**) Boxplots illustrate the ratio of newly identified transcript variants in relation to all identified transcripts in each tissue. The median ratio exhibited a range from 0.46 to 0.59. Tissues, representing the minimum and maximum ratio within 1.5 times the Interquartile range, are displayed in the plot. Outlier tissues identified below the minimum whisker include the following: 0.0001 TPM: brain, brain opioid, brain Alzheimer, brain fetal; 0.001 TPM: brain, brain opioid, brain Alzheimer, brain fetal; 0.01 TPM: brain, brain opioid, brain Alzheimer. (**B**) Spearman correlations of ratios against number of samples per tissue. The respective linear regression curves with the 95% confidence interval are given.

| 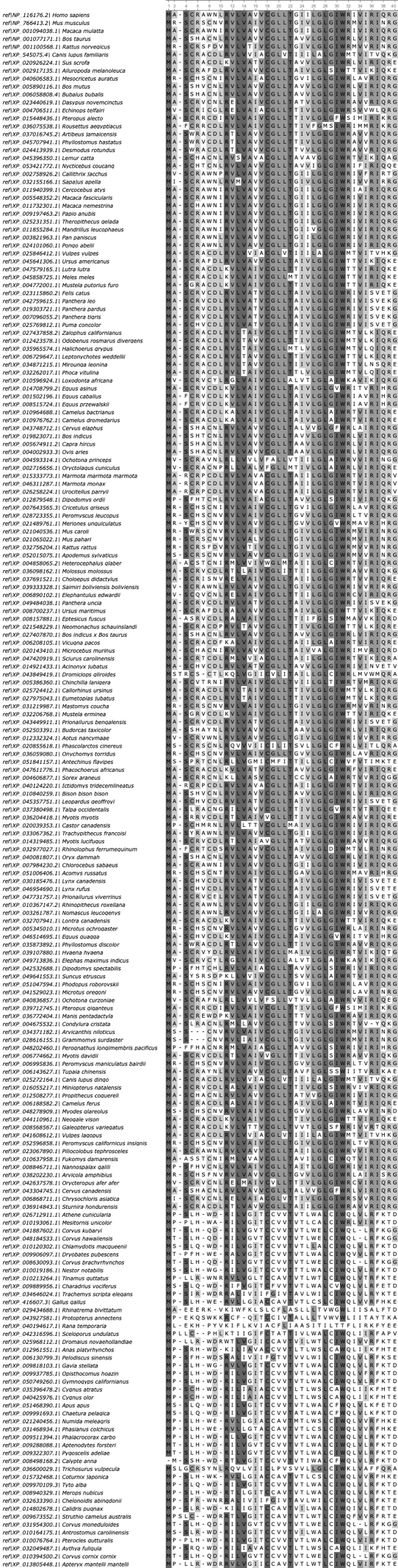 | **Figure S4. Amino acid sequence alignment of the first transmembrane helix of ADGRG7/GPR128.** The amino acid sequences of vertebrate orthologs of ADGRG7/GPR128 were aligned using Uniprot Ugene [(3)](https://www.zotero.org/google-docs/?wQWZ3t) and the implemented MUSCLE algorithm with default settings. The species names and the NCBI identifier of the protein sequences are given. |
| --- | --- |

# **
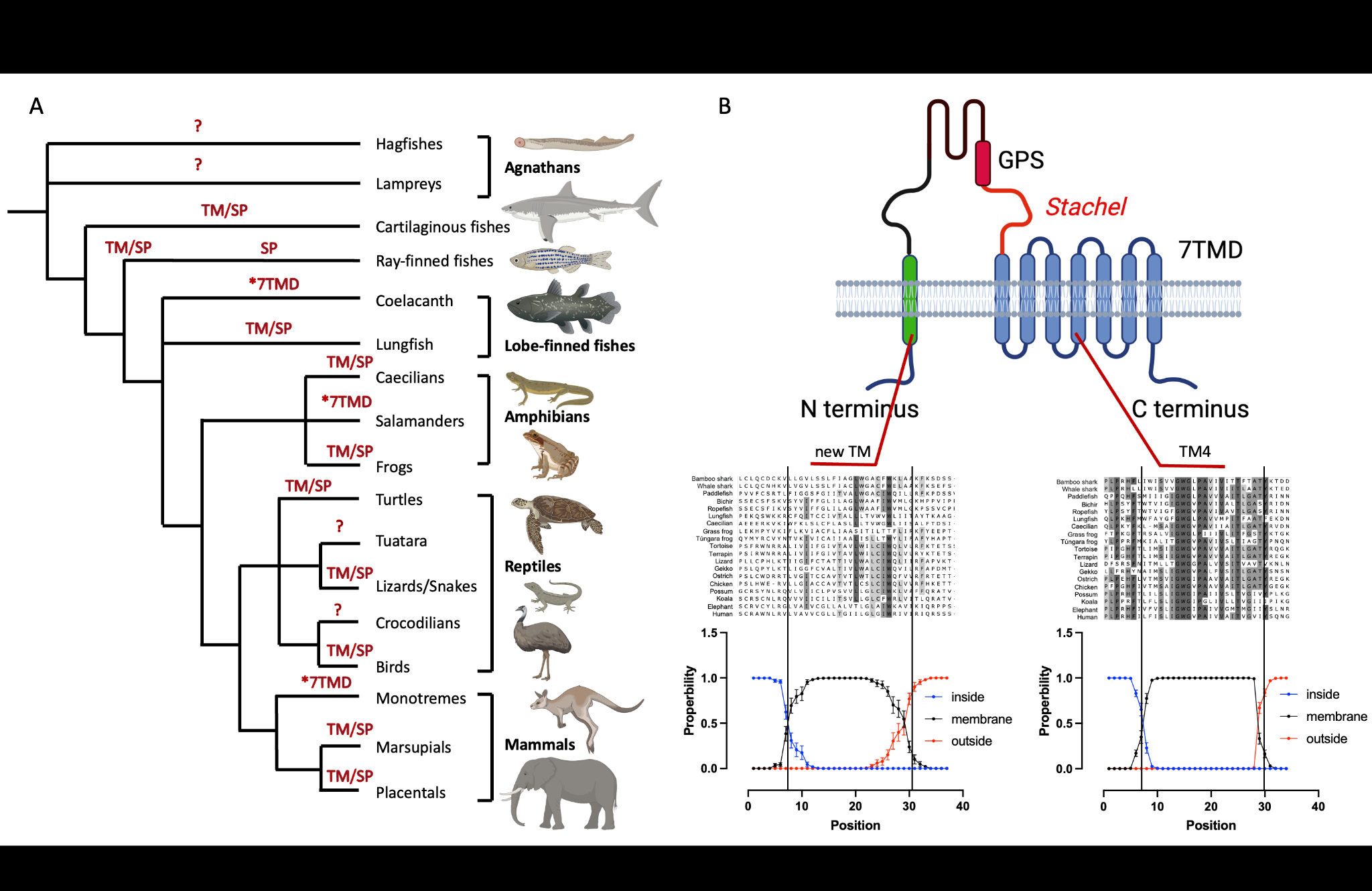
**

**Figure S5. The transmembrane helix at the very N terminus is present in most vertebrate ADGRG7/GPR128 orthologs.** (**A**) The phylogenetic analysis of currently available sequence data from NCBI (date: 2023/11/09) revealed that ADGRG7/GPR128 is present in all vertebrate classes at least with partial sequences (*7TMD) but not yet found in agnathans and crocodiles (marked with ?). Moreover, the newly identified transmembrane helix (TM) at the very N terminus of ADGRG7/GPR128 is present in sharks (bamboo shark (*Chiloscyllium plagiosum*), whale shark (*Rhincodon typus*)) and very basal ray-finned fishes (American paddlefish (*Polyodon spathula*), Senegal bichir (*Polypterus senegalus*), ropefish (*Erpetoichthys calabaricus*)) but not in other ray-finned fishes where the receptor sequence usually starts with a signal peptide sequence (SP). Many vertebrates have transcript variants encoding either the TM or the SP (TP/SP). The newly identified transmembrane helix is continuously found in lobe-finned fishes (West African lungfish (*Protopterus annectens*)) and many amphibians, reptiles, and mammals. (**B**) The amino acid sequences of the new transmembrane helix (new TM) and, for comparison purposes, the transmembrane helix 4 (TM4) of the 7TMD representatives from most vertebrate classes were aligned and the helix probability scores (mean ± SEM) of the individual sequences per position (DeepTMHMM version 1.0.24) are plotted (n=19 species). The complete sequences and accession numbers are available in the fasta file: vertebrate_ADGRG7.fasta (Supplementary File S1).

**
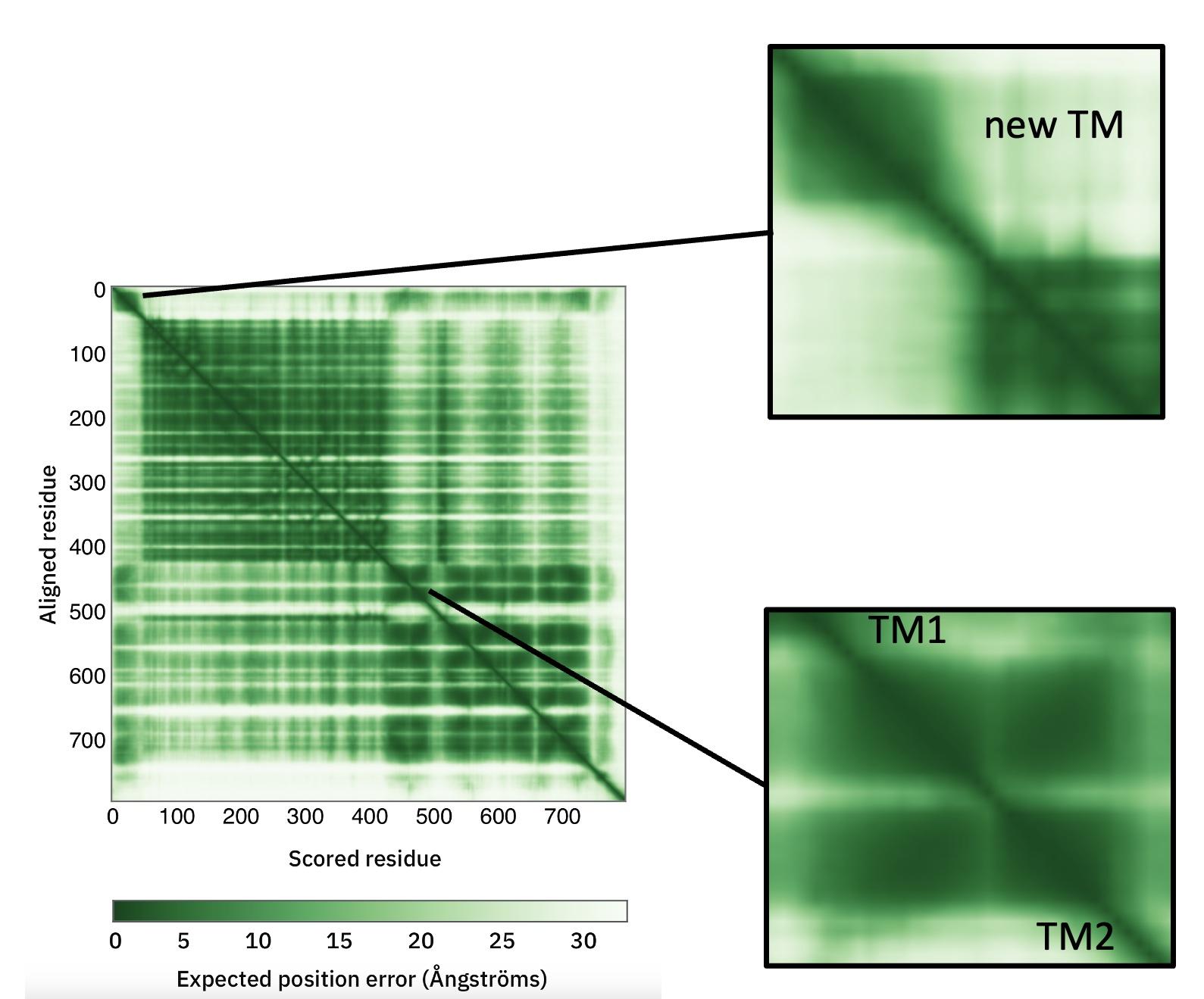
**

**Figure S6. Predicted alignment error (PAE) analysis of the AlphaFold2 models of the human ADGRG7/GPR128.** The PAE values of every position to all other positions within the AlphaFold2 model of the human ADGRG7 (AF-Q96K78-F1-model_v4) are plotted. The PAE of the new TM and the TM1 and TM2 of the 7TMD are enlarged.

# **Supplementary Tables**

Please note that large tables (Supplementary Tables S2-S7) have been placed in a separate spreadsheet.

**Table legends**

**Table S1. RNA-seq datasets used.** This document.

**Table S2. Tissues included in the analysis.**

**Table S3. Comparison of identified transcript variants in Splice-O-Mat and GTEx/NCBI.**

**Table S4. Comparison of identified transcript variants/exons between human and mouse data from Knierim *et al.* 2019.**

**Table S5. Expression of the detected transcript variants for all 33 aGPCRs in all tissues (TPM)**.

**Table S6. Quantification of overall expression of all 33 aGPCR in 48 different tissue types, sum of all transcript variants in TPM.**

**Table S7. Identified exons within new transcript variants that were not part of diagnostic exome sequencing or gnomAD.**

**Table S1.** RNA-seq datasets. Accession number of RNA-seq datasets used for transcript assembly. Tissue type, sample size, read length, sequencing depth (reads per million) and library preparation of the RNA-seq data are listed. The origin of the samples and the original citations, if available, are given.

| **Dataset** | **Tissue** | **Sample size** | **Read length** | **Reads per Sample** | **Description** | **Library prep** | **Citation** |
| --- | --- | --- | --- | --- | --- | --- | --- |
| GSE173955 | brain | 40 | 101 | 9-25M | postmortem Alzheimer’s disease (AD) brains, 8 AD and 10 non-AD | random primers, Illumina TruSeq stranded mRNA LT Sample Prep kit. | [(4)](https://www.zotero.org/google-docs/?YRJlbM) |
| GSE182321 | brain | 41 | 150 | 27-34M | 29 Opioid use disorder (OUD) and 18 non-psychiatric controls, 27 OUD subjects and 14 controls were used to generate ribonucleic acid (RNA) sequencing data (RNAseq) | random primers, NEBNext® Ultra™ RNA Library Prep Kit | [(5)](https://www.zotero.org/google-docs/?2ZYof3) |
| GSE101521 | brain | 59 | 101 | 7-61M | on-psychiatric controls (CON, N=29), DSM-IV major depressive disorder suicides (MDD-S, N=21) and MDD non-suicides (MDD, N=9) in the dorsal lateral prefrontal cortex (Brodmann Area 9) | random primers, TruSeq Stranded Total RNA Sample Prep kit (Illumina, San Diego, CA, USA) | [(6)](https://www.zotero.org/google-docs/?tnCfLo) |
| GSE174478 | liver | 94 | 101 | 31-44M | a fatty liver diagnosed ultrasonically by an increase in hepatorenal contrast, a history of alcohol consumption of less than 30 g/d for men and less than 20 g/d for women, seronegativity for hepatitis B virus surface antigen and hepatitis C virus antibody, and the absence of autoimmune hepatitis, primary biliary cholangitis, primary sclerosing cholangitis, Budd-Chiari syndrome, Wilson disease, and drug-induced liver injury | random primers, TruSeq Stranded Total RNA LT Sample Prep Kit | [(7)](https://www.zotero.org/google-docs/?Q2WaaU) |
| GSE217427 | kidney | 44 | 50 | 37-51M | medulla and coretex, with human kidney damage (KD) (n=22) and without KD (22) | random primer, library kit KAPA RNA HyperPrep Kit with RiboErase (Roche) | Not published yet |
| GSE165303, SRP302848 | heart | 101 | 150 | 57-80M | with dilated cardiomyopathy, 50 non-failing, 2 Transfected with control adenovirus and 2 transfected with HAND1 overexpressing adenovirus, **only paired end was selected** | random primers | Not published yet |
| GSE138734, SRP225193 | many tissues | 457 | 76 | 76-111M | 300 human samples, including 45 tissues, 162 cell types, and 93 cell lines, some paired some single end, total RNA (296 samples), **only full RNA-seq (paired end) of 45 tissues was selected (cell line and cell types excluded)** | random primers, TruSeq stranded total-RNA library prep kit with Ribo-Zero Gold | [(8)](https://www.zotero.org/google-docs/?QKPEAg) |
| PRJEB23709 at ENA | melanoma | 91 | 101 | around 50M | RNA-seq of metastatic melanoma patients treated with anti-PD-1 alone or combined anti-PD-1 and anti-CTLA-4 immunotherapy | random primers, TruSeq RNA Access Library Prep Kit = TruSeq RNA Exome, RNA coding regions | [(9)](https://www.zotero.org/google-docs/?5kmC0X) |

# **Supplementary References**

[1. Dobin,A. and Gingeras,T.R. (2016) Optimizing RNA-Seq Mapping with STAR. *Methods Mol. Biol. Clifton NJ*, **1415**, 245–262.](https://www.zotero.org/google-docs/?bbbUrf)

[2. Pertea,M., Pertea,G.M., Antonescu,C.M., Chang,T.-C., Mendell,J.T. and Salzberg,S.L. (2015) StringTie enables improved reconstruction of a transcriptome from RNA-seq reads. *Nat. Biotechnol.*, **33**, 290.](https://www.zotero.org/google-docs/?bbbUrf)

[3. Okonechnikov,K., Golosova,O., Fursov,M., and the UGENE team (2012) Unipro UGENE: a unified bioinformatics toolkit. *Bioinformatics*, **28**, 1166–1167.](https://www.zotero.org/google-docs/?bbbUrf)

[4. Hokama,M., Oka,S., Leon,J., Ninomiya,T., Honda,H., Sasaki,K., Iwaki,T., Ohara,T., Sasaki,T., LaFerla,F.M., *et al.* (2014) Altered expression of diabetes-related genes in Alzheimer’s disease brains: the Hisayama study. *Cereb. Cortex N. Y. N 1991*, **24**, 2476–2488.](https://www.zotero.org/google-docs/?bbbUrf)

[5. Mendez,E.F., Wei,H., Hu,R., Stertz,L., Fries,G.R., Wu,X., Najera,K.E., Monterey,M.D., Lincoln,C.M., Kim,J.-W., *et al.* (2021) Angiogenic gene networks are dysregulated in opioid use disorder: evidence from multi-omics and imaging of postmortem human brain. *Mol. Psychiatry*, **26**, 7803–7812.](https://www.zotero.org/google-docs/?bbbUrf)

[6. Pantazatos,S.P., Huang,Y.-Y., Rosoklija,G.B., Dwork,A.J., Arango,V. and Mann,J.J. (2017) Whole-transcriptome brain expression and exon-usage profiling in major depression and suicide: evidence for altered glial, endothelial and ATPase activity. *Mol. Psychiatry*, **22**, 760–773.](https://www.zotero.org/google-docs/?bbbUrf)

[7. Kawamura,S., Matsushita,Y., Kurosaki,S., Tange,M., Fujiwara,N., Hayata,Y., Hayakawa,Y., Suzuki,N., Hata,M., Tsuboi,M., *et al.* (2022) Inhibiting SCAP/SREBP exacerbates liver injury and carcinogenesis in murine nonalcoholic steatohepatitis. *J. Clin. Invest.*, **132**, e151895.](https://www.zotero.org/google-docs/?bbbUrf)

[8. Lorenzi,L., Chiu,H.-S., Avila Cobos,F., Gross,S., Volders,P.-J., Cannoodt,R., Nuytens,J., Vanderheyden,K., Anckaert,J., Lefever,S., *et al.* (2021) The RNA Atlas expands the catalog of human non-coding RNAs. *Nat. Biotechnol.*, **39**, 1453–1465.](https://www.zotero.org/google-docs/?bbbUrf)

[9. Gide,T.N., Quek,C., Menzies,A.M., Tasker,A.T., Shang,P., Holst,J., Madore,J., Lim,S.Y., Velickovic,R., Wongchenko,M., *et al.* (2019) Distinct Immune Cell Populations Define Response to Anti-PD-1 Monotherapy and Anti-PD-1/Anti-CTLA-4 Combined Therapy. *Cancer Cell*, **35**, 238-255.e6.](https://www.zotero.org/google-docs/?bbbUrf)
